# Maternal pre-pregnancy BMI and gestational weight gain, offspring DNA methylation and later offspring adiposity: findings from the Avon Longitudinal Study of Parents and Children

**DOI:** 10.1093/ije/dyv042

**Published:** 2015-04-08

**Authors:** Gemma C Sharp, Debbie A Lawlor, Rebecca C Richmond, Abigail Fraser, Andrew Simpkin, Matthew Suderman, Hashem A Shihab, Oliver Lyttleton, Wendy McArdle, Susan M Ring, Tom R Gaunt, George Davey Smith, Caroline L Relton

**Affiliations:** ^1^MRC Integrative Epidemiology Unit at the University of Bristol, Bristol, UK,; ^2^School of Social and Community Medicine, University of Bristol, Bristol, UK and; ^3^Institute of Genetic Medicine, University of Newcastle, Newcastle upon Tyne, UK

**Keywords:** Epigenetic, ALSPAC, ARIES, causality, epigenome-wide association study, longitudinal, overweight, overnutrition, undernutrition

## Abstract

**Background:** Evidence suggests that *in utero* exposure to undernutrition and overnutrition might affect adiposity in later life. Epigenetic modification is suggested as a plausible mediating mechanism.

**Methods:** We used multivariable linear regression and a negative control design to examine offspring epigenome-wide DNA methylation in relation to maternal and offspring adiposity in 1018 participants.

**Results:** Compared with neonatal offspring of normal weight mothers, 28 and 1621 CpG sites were differentially methylated in offspring of obese and underweight mothers, respectively [false discovert rate (FDR)-corrected *P*-value < 0.05), with no overlap in the sites that maternal obesity and underweight relate to. A positive association, where higher methylation is associated with a body mass index (BMI) outside the normal range, was seen at 78.6% of the sites associated with obesity and 87.9% of the sites associated with underweight. Associations of maternal obesity with offspring methylation were stronger than associations of paternal obesity, supporting an intrauterine mechanism. There were no consistent associations of gestational weight gain with offspring DNA methylation. In general, sites that were hypermethylated in association with maternal obesity or hypomethylated in association with maternal underweight tended to be positively associated with offspring adiposity, and sites hypomethylated in association with maternal obesity or hypermethylated in association with maternal underweight tended to be inversely associated with offspring adiposity.

**Conclusions:** Our data suggest that both maternal obesity and, to a larger degree, underweight affect the neonatal epigenome via an intrauterine mechanism, but weight gain during pregnancy has little effect. We found some evidence that associations of maternal underweight with lower offspring adiposity and maternal obesity with greater offspring adiposity may be mediated via increased DNA methylation.

**Key Messages**

- This study identified several CpG sites that are differentially methylated in the cord blood of offspring of obese or underweight mothers compared with offspring of normal weight mothers, with no overlap in the sites that maternal obesity and underweight relate to. The comparison of underweight with normal weight mothers identified many more differentially methylated sites (1621) than the comparison of obese with normal weight mothers (28), suggesting that maternal underweight has a larger effect on the fetal epigenome than maternal obesity does. At most sites, maternal body mass index outside the normal range was associated with higher offspring methylation. The effect of maternal obesity was stronger than the effect of paternal obesity, supporting an underlying intrauterine mechanism.
- There were no consistent associations of gestational weight gain with DNA methylation in this study.
- In general, sites that were hypermethylated in association with maternal obesity or hypomethylated in association with maternal underweight tended to be positively associated with offspring adiposity, and sites hypomethylated in association with maternal obesity or hypermethylated in association with maternal underweight tended to be inversely associated with offspring adiposity. This raises the possibility that associations of maternal and offspring adiposity, particularly intergenerational persistence of the maternal phenotype, may be mediated by DNA methylation.
- We sought external validation by comparing our associations with those previously reported in the literature. We identified four studies reporting associations at four loci, but we did not consistently replicate the direction or the magnitude of these associations.

## Introduction

Evidence from observational studies where pregnant women have experienced extreme nutritional deficiency suggests that *in utero* exposure to such undernutrition might increase the risk of later-life adiposity in the offspring.^1^ At the other extreme, greater maternal body mass index (BMI) at the start of pregnancy and greater gestational weight gain (GWG) have also been found to be associated with greater subsequent offspring adiposity.^2–4^ Within-sibling studies^5^^,^^6^ and some^7^^,^^8^ though not all^9^^,^^10^ before and after bariatric surgery studies suggest that in cases of extreme maternal adiposity these associations might be causal via an intrauterine mechanism. Together, these results suggest that exposure to both undernutrition and overnutrition *in utero* might result in greater adiposity in later life.

The intrauterine mechanism through which maternal nutrition might influence long-term offspring adiposity is unclear. Increasingly, epigenetic modification is suggested as a plausible mediating causal mechanism,^3^^,^^11–13^ but evidence for such mediation in human studies is limited. Epigenetic modifications can cause stable changes to gene expression and cellular phenotype without altering the underlying genome. For example, DNA methylation, which is the most widely-studied epigenetic modification, involves the addition of methyl groups to DNA at CpG sites—where cytosine occurs next to guanine in the nucleotide sequence. Four studies, two examining famine experienced during the Dutch Hunger Winter^14^^,^^15^ and two examining seasonal nutritional variation in the Gambia,^16^^,^^17^ have reported differential DNA methylation in offspring exposed to maternal undernutrition during fetal life compared with those who were not exposed *in utero* to maternal undernutrition. At the other extreme of the BMI distribution, one study found 5698 differentially methylated CpG sites in the peripheral blood of children born to morbidly obese mothers prior to bariatric surgery compared with their siblings born after maternal bariatric surgery and associated weight loss.^18^ Others studies have found weak or no evidence of associations between maternal BMI and offspring DNA methylation.^19–23^ Only two studies have investigated the relationship between DNA methylation at birth and adiposity in later childhood. Both studies used a candidate-gene approach and each identified a single different associated locus.^24^^,^^25^

Thus existing evidence is mixed, and several limitations make drawing conclusions difficult. Specifically: (i) given the evidence that both maternal undernutrition and overnutrition in pregnancy might influence offspring adiposity, there may be non-linear associations of the maternal exposures with offspring DNA methylation, which, with one exception,^22^ previous studies have not explored; (ii) only one study^24^ has looked at the potential mediating role of methylation between exposure and outcome (i.e. maternal adiposity in relation to offspring DNA methylation and offspring DNA methylation in relation to offspring adiposity outcome) in a single study; (iii) only one study^20^ has looked at whether associations between maternal adiposity and offspring DNA methylation persist over time (i.e. as offspring age), which might be relevant to fully understanding mediation mechanisms; and (iv) no study has applied a causal analysis framework to explore the likelihood of epigenetic mediation being a truly causal process in this context, independent of confounding or other sources of bias.

**Figure 1.**
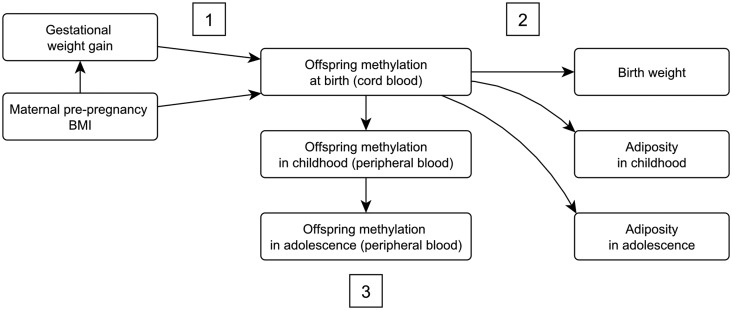
A conceptual framework showing the relationships of interest. GWG and maternal BMI are hypothesized to affect offspring adiposity via offspring DNA methylation. Specific hypotheses are: (i) maternal pre-pregnancy BMI and/or GWG is associated with differential methylation at CpG sites in offspring cord blood; (ii) methylation at these sites is also associated with offspring adiposity (we would also expect consistency between different measures of adiposity and age at measurement); and (iii) maternal adiposity is persistently associated with differential methylation in offspring at time points beyond birth.

In this paper, our primary aim is to identify differential methylation in offspring at birth that is associated with both maternal adiposity in pregnancy and offspring adiposity from birth to adolescence. Figure 1 shows the conceptual framework and hypothesized relationships. We hypothesize that: (i) maternal pre-pregnancy BMI and/or GWG are associated with differential methylation at CpG sites in offspring cord blood; (ii) methylation at these sites is also associated with offspring adiposity (we would also expect consistency between different measures of adiposity and age at measurement); and (iii) maternal adiposity is persistently associated with differential methylation in offspring at time points beyond birth. We addressed some previous limitations in human studies by: (i) testing non-linear associations of maternal pre-pregnancy BMI or GWG with DNA methylation; (ii) examining associations of maternal exposures with offspring methylation at different ages and then examining whether identified differentially methylated sites are related to subsequent offspring adiposity; and (iii) using a negative control (paternal BMI)^26^ to explore causation.^27^

## Methods

### Participants

The Avon Longitudinal Study of Parents and Children (ALSPAC) is a general population pregnancy cohort study that initially recruited 14 541 pregnancies with a due date between April 1991 and December 1992 in Avon, UK.^28^^,^^29^ Written informed consent has been obtained for all ALSPAC participants. Ethical approval was granted from the ALSPAC Law and Ethics Committee and the local Research Ethics Committees. Please note that the study website contains details of all the data that are available through a fully searchable data dictionary [http://www.bris.ac.uk/alspac/researchers/data-access/data-dictionary/]. Blood samples were collected from all consenting ALSPAC mothers and their offspring at several clinics at different time points. DNA methylation data for a subset of 1018 mother-offspring pairs was generated as part of the Accessible Resource for Integrated Epigenomics Studies (ARIES) project [http://www.ariesepigenomics.org.uk/]. ARIES participants were selected based on availability of DNA samples at two time points for the mother (antenatal and at follow-up when the offspring were mean age 17.1 years) and three time points for the offspring (neonatal, childhood, mean age 7.5 and adolescence, mean age 17.1 years). For this study, we used methylation data for 914 mother-neonate, 973 mother-child and 974 mother-adolescent pairs for whom data on all variables of interest were available.

### Measures of maternal and paternal adiposity

#### Pre-pregnancy BMI

Maternal pre-pregnancy weight and height were self-reported, and paternal weight and height were reported by the mother. Data for partners who were not confirmed as being the biological father of the child by the mothers’ report were excluded. In order to explore the possibility that systematic bias in partner or self-report weight (e.g. those who are heavier systematically under-reporting their weight) might bias our findings, we used Bland-Altman plots (plots of mean and difference in mean), which suggested that the level of misreporting is similar for the majority of participants and is not markedly influenced by mean weight (more detailed results and plots available as Supplementary data at *IJE* online). BMI was calculated as weight (kg)/height (m^2^) and categorized according to World Health Organization categories (underweight <18.5 kg/m^2^; normal 18.5–24.9 kg/m^2^; overweight 25.0–29.9 kg/m^2^; obese ≥ 30.0 kg/m^2^).

#### Gestational weight gain

Six trained research midwives abstracted data from obstetric medical records.^2^^,^^30^ These data included every weight measurement made in antenatal clinics during pregnancy (median 10 measures per woman, interquartile range: 8 to 11) and the corresponding gestational age and date, as previously described. Multi-level models were used to derive the estimated pre-pregnancy weight and GWG per week in different periods of pregnancy from these repeat measurements as previously described.^2^^,^^30^

We assessed associations with GWG in three ways:

- Total weight change defined as the last weight minus the first weight measurement in pregnancy.
- According to Institute of Medicine (IoM) recommendations,^31^ women were categorized as having adequate, less than recommended and excessive GWG. Using these recommendations, adequate total GWG as: 12.5–18 kg for underweight women (<18.5 kg/m^2^); 11.5–16 kg for normal weight women (18.5–24.9 kg/m^2^); 7–11.5 kg for overweight women (25–29.9 kg/m^2^); and 5–9 kg for obese women (>30 kg/m^2^), with women gaining less that these ranges within each BMI category defined as having inadequate GWG and those gaining more as having excessive GWG.
- Rate of weight gain per week in early-pregnancy (between 0 and 18 weeks), mid pregnancy (between 18 and 28 weeks) and late pregnancy (between 28 weeks and birth) calculated using a multi-level spline model, as previously reported.^2^^,^^30^ These models were limited to term (≥37 weeks of gestation) pregnancies.

### Measures of offspring adiposity

Birthweight was extracted from medical records. Height in childhood [mean age 7.5 years (SD: 0.2)] and adolescence [mean age 15.5 years (SD: 0.4)] was measured using a standard protocol to the last complete millimetre using the Harpenden Stadiometer (Holtain Crosswell, Dyfed, UK). Weight was measured to the nearest 50 g using the Tanita Body Fat Analyser (Model TBF 305, Tanita, Arlington Heights, IL). BMI was calculated as weight (kg)/height (m^2^). Waist circumference in childhood and adolescence (mean ages as above) were measured to the nearest millimetre at the minimum circumference of the abdomen between the iliac crests and the lowest ribs, and the tape was kept perpendicular to the long axis of the body, touching the skin but not compressing the tissue. Fat and lean mass (g) were assessed by whole-body dual-energy X-ray absorptiometry (DXA) (Prodigy scanner, Lunar Radiation Corp, Madison, WI, USA) at a mean age of 9.9 years (SD: 0.3) and again at age 15.5 (SD: 0.4).

### Methylation data

Full details of the collection and generation of DNA methylation data are available as Supplementary data at *IJE* online.

DNA methylation in cord or peripheral blood was measured using the Illumina Infinium® HumanMethylation 450 K BeadChip assay at the University of Bristol as part of the ARIES project [ariesepigenomics.org.uk]. The Illumina Infinium® HumanMethylation 450 K BeadChip platform can detect between-group differences of 20% methylation with a lower than 1% false-positive rate.^32^ Data were pre-processed in R (version 3.0.1) with the WateRmelon package^33^ according to the subset quantile normalization approach described by Touleimat and Tost^34^ in an attempt to reduce the non-biological differences between probes. Allocation to bisulfite conversion batch (96-well plate) was identified as the major batch variable likely to contain systematic bias (Figure S1, available as Supplementary data at *IJE* online) so this was controlled for in all analyses.

### Other variables

Maternal age at delivery was derived from the mother’s report of her own and her baby’s dates of birth. Maternal occupation was classified according to the 1991 British Office of Population and Census Statistics classification and categorized for this study as manual or non-manual. Parity was extracted from medical records and categorized for this study as nulliparous or parous; maternal smoking behaviour was assessed during pregnancy via questionnaire and categorized for this study as: (i) never smoking during pregnancy; (ii) smoking before pregnancy or in the first trimester and then stopping; or (iii) smoking throughout pregnancy.

Cell-type proportions were estimated using the estimateCellCounts function in the minfi R package,^35^ which is based on the method developed by Houseman.^36^ This estimated the proportion of B cells, CD8 T cells, CD4 Tcells, granulocytes, NK cells and monocytes in each sample.

### Statistical analysis

We compared mothers in ARIES with those in the rest of ALSPAC by calculating means and standard deviations for key characteristics. We used linear regression to confirm that the associations between maternal adiposity and offspring adiposity that have been described in other cohorts, including ALSPAC,^2^^,^^37^^,^^38^ were also present in the ARIES subset. We used linear regression to investigate associations between maternal adiposity (exposure) and estimated cell-type proportions in cord blood (outcome).

In order to ensure our analyses were appropriate and robust, we explored the impact on our results of logit-transforming methylation β-values to ‘M-values’.^39^ As specified in the introduction, we hypothesized that any association between maternal adiposity and offspring methylation might be non-linear. We tested this in two ways: (i) examining associations of maternal BMI categories (defined using WHO criteria) with epigenome-wide methylation, comparing offspring methylation in offspring from underweight, overweight and obese mothers with those from normal weight mothers; and (ii) examining evidence of departure from linearity with continuously measured BMI and total and stage-specific GWG by adding a quadratic term to models with these exposures. We also tested the hypothesis that there may be an interaction between continuous pre-pregnancy BMI and total GWG or stage-specific GWG/week in their association with epigenome-wide offspring methylation. Full details of these analyses are available as Supplementary data at *IJE* online (EWAS Regression Model Optimization).

Epigenome-wide association studies (EWAS) were conducted in R version 3.0.1. We used multiple linear regression to model maternal pre-pregnancy BMI or GWG as the exposure and methylation (untransformed β-values) as the outcome. Bisulfite conversion batch (96-well plate) was included in all models as a fixed effect (rather than random effect) to reduce computational intensity. Linear regression was applied to each CpG site individually and results were then corrected for multiple testing by controlling the expected proportion of false-positives among all discoveries (FDR) using the Benjamini–Hochberg method. The genomic inflation factor (Lambda) and quantile-quantile (Q-Q) plots were used to compare the genome-wide distribution of *P*-values with the expected null distribution (Figure S2, available as Supplementary data at *IJE* online).

We examined three models: Model 1 adjusted for bisulfite conversion batch only; Model 2 adjusted for batch and covariates: offspring sex, maternal age, parity, smoking status, occupation, gestational age at birth (only in analyses where gestational age at birth was not considered to be on the proposed causal pathway and the exposure measure was not derived using gestational age at birth—i.e. total weight gain and IoM-recommended GWG), pre-pregnancy BMI (where the exposure was not derived using pre-pregnancy BMI—i.e. total GWG or pregnancy stage-specific GWG) and weight change in any previous stages of pregnancy (only in pregnancy stage-specific GWG); and Model 3 adjusted for batch, covariates as above and estimated cell-type proportions.

EWAS results were summarized as mean (and 95% confidence interval) differences in percentage methylation (Δβ*100) per unit exposure for continuous variables and between groups for binary categorical variables. EWAS were conducted using methylation data collected at three time-points – birth, childhood and adolescence.

Cord blood CpG sites associated with maternal adiposity with an FDR-adjusted *P*-value < 0.05 were taken forward to be assessed in a longitudinal framework. A multilevel model^40^^,^^41^ including a random intercept and a linear regression spline term to allow for flexibility was fitted to each of these sites sequentially. This model is described in more detail in the supplementary material available as Supplementary data at *IJE* online (Longitudinal Model). Models were adjusted for confounders (offspring sex, maternal age, parity, smoking status and occupation) and the first 20 independent surrogate variable components (which account for cellular heterogeneity between the cord blood and peripheral blood cells). To correct for multiple testing, across the CpG sites and two parameters of (difference in change during childhood/adolescence) interest we used a cut-off of 0.05/(2*number of CpG sites).

Cord blood CpG sites associated with maternal adiposity with an FDR-adjusted *P*-value < 0.05 were also taken forward to be assessed in relation to offspring adiposity using linear regression. We adjusted for bisulfite conversion batch, sex and age at measurement (months). Where birthweight was the outcome, age was defined as gestational age at birth. Where waist circumference, fat or lean mass were the outcomes, we additionally adjusted for height (cm) at measurement. Results were corrected for multiple testing using the FDR method and summarized as mean (and 95% confidence interval) difference in offspring adiposity per 10% change in methylation level.

### Replication and assessing robustness of findings

We assessed the robustness of our findings in two ways. Firstly, for cord blood CpG sites that were associated with maternal adiposity (FDR-adjusted *P*-value < 0.05) we used a negative control study to explore causality by comparing these associations with those of paternal adiposity with cord blood methylation.^26^ Similar associations for maternal and paternal adiposity with offspring outcomes would suggest that associations are explained by confounding by shared familial exposures, whereas a stronger maternal association would support a direct intrauterine effect.^26^ Additionally, we systematically searched the literature for all published studies that had examined maternal BMI and/or GWG with cord blood DNA methylation and compared our findings with findings from those studies.

## Results

### ARIES sample characteristics

Table S1 (available as Supplementary data at *IJE* online) compares the characteristics of mothers in ARIES with those in the rest of ALSPAC. Mothers included in ARIES were slightly older, more likely to have a non-manual occupation and less likely to have smoked throughout pregnancy.

We confirmed that, in the ARIES subset, maternal pre-pregnancy BMI was positively associated with measures of offspring adiposity as previously found for the whole cohort with relevant data^2^ (Table S2, available as Supplementary data at *IJE* online). In line with this linear trend, maternal underweight was inversely associated with offspring adiposity whereas maternal overweight and, to a greater extent, obesity were positively associated with offspring adiposity. GWG in early and mid pregnancy were associated with birthweight, but associations with other measures of childhood adiposity were mostly limited to late GWG (Table S2, available as Supplementary data at *IJE* online).

There were few associations between estimated cell-type proportions and measures of maternal adiposity (Table S3, available as Supplementary data at *IJE* online), suggesting that our EWAS results are unlikely to be explained by confounding by cell-type variation. Partly for this reason, and partly because the estimated cell proportions method uses reference data derived from adult whole blood that may not be appropriate for cord blood analysis,^36^ we favour and therefore report the EWAS results of Model 2 (adjusted for bisulfite conversion batch and maternal confounders) over Model 1 (adjusted for bisulfite conversion batch only) and Model 3 (adjusted for bisulfite conversion batch, maternal confounders and estimated cell-type proportions). File S1 (available as Supplementary data at *IJE* online) presents the results from all three models and shows that results are generally similar in the three models.

After empirical optimization of our regression models (see Supplementary data available at *IJE* online: EWAS regression model optimization) in which we confirmed that the assumption of a linear relationship between BMI/GWG and cord blood DNA methylation is valid [for 94.2–100% of probes tested, the model fit was not improved (likelihood ratio test *P*-value > 0.05) by the inclusion of a quadratic term for pre-pregnancy BMI/GWG], we carried out EWAS of offspring methylation regressed on pre-pregnancy BMI or GWG. Figure S2 (available as Supplementary data at *IJE* online) shows genomic inflation values (Lambdas) and quantile-quantile (Q-Q) plots for each EWAS conducted using Model 2. The numbers of CpG sites identified by each analysis are provided in Table S4, and File S1 lists all results with an FDR-adjusted *P*-value < 0.05 for each model (available as Supplementary data at *IJE* online).

### Maternal pre-pregnancy BMI and offspring methylation at birth

As a continuous variable, maternal pre-pregnancy BMI (*n* = 727) was positively associated with cord blood methylation at two CpG sites, one at *CCDC112* (cg13013671 on the gene body 200 base pairs from a transcription start site) and one at *MCOLN3* (cg05003422 at a 5′ untranslated region), but the effect sizes were small [0.11 (0.07, 0.015) and 0.02 (0.02, 0.03) change in percentage methylation per 1 kg/m^2^ change in BMI, respectively].

**Figure 2.**
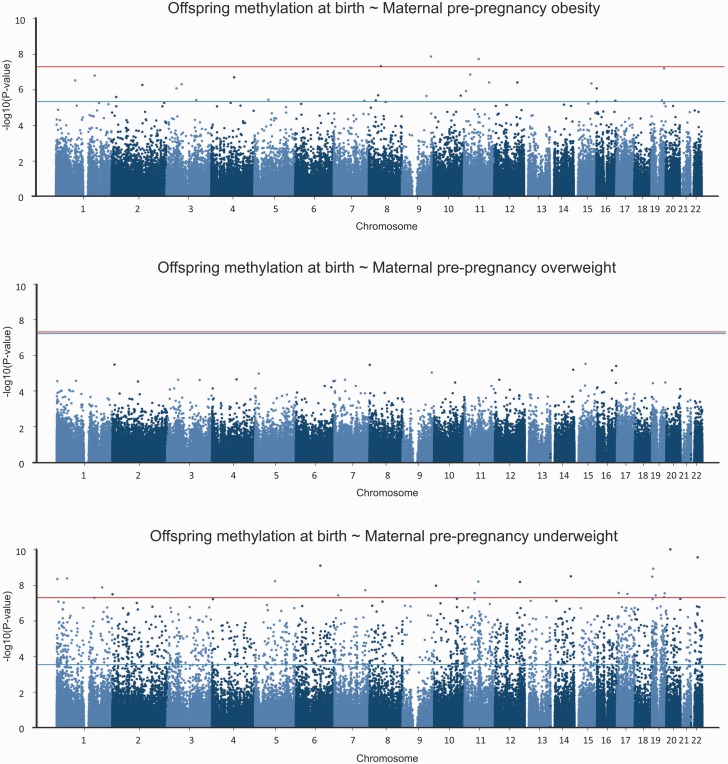
Manhattan plots showing the results of epigenome-wide association studies (EWAS) of cord blood DNA methylation in offspring of underweight (*n* = 24), overweight (n = 94) and obese (*n* = 32) mothers compared with offspring of normal weight mothers (*n* = 577). The bottom (blue) line indicates the FDR-adjusted *P*-value threshold (0.05) and the top (red) line indicates the Bonferroni threshold for genome-wide significance (3.5*10^−7^, i.e. 0.05/284972 probes).

Compared with offspring of women who were normal weight (*n* = 577), offspring of women who were obese (*n* = 32) had 28 sites that were differentially methylated and offspring of women who were underweight (n=24) had a considerably larger number (1621) of sites that were differentially methylated (FDR-corrected *P*-value < 0.05; Figure 2). Lambdas did not suggest genomic inflation (Figure S2, available as Supplementary data at *IJE* online: 0.998 for maternal obesity and 0.958 for maternal underweight). There was no overlap in terms of sites associated with maternal obesity and sites associated with maternal underweight. A positive association, where higher methylation is associated with BMI outside the normal range, was seen at 78.6% of the sites associated with obesity and 87.9% of the sites associated with underweight. Table 1 summarizes the top five sites with the largest effect sizes and FDR-adjusted *P*-values < 0.05. There were no sites that were differentially methylated in offspring of overweight women (*n* = 94) compared with normal weight women (Figure 2).

**Table 1.** The top five CpG sites with the largest effect size and FDR-adjusted *P*-value < 0.05 for associations between maternal pre-pregnancy obesity or underweight and offspring cord blood methylation

Exposure	Gene region (CpG)	CHR	Gene feature group	Relation to CpG Island	β (95% CI)*	FDR-adjusted P-value
Pre-pregnancy maternal obesity	unnamed (cg00526953)	16		North shore	−14.99 (−20.89, −9.1)	0.016
	*SUCLG2* (cg02321096)	3	3'UTR		−11.4 (−15.78, −7.02)	0.012
	*FAM129B* (cg03270036)	9	Gene body	North shore	−9.65 (−12.92, −6.37)	0.003
	*KIF15;KIAA1143* (cg17546649)	3	TSS1500; Gene body	North shore	7.6 (4.61, 10.59)	0.016
	*STAB2* (cg23131355)	12	TSS1500		−5.05 (−6.98, −3.12)	0.011
Pre-pregnancy maternal underweight	*TEX14* (cg01796478)	17	5'UTR	Island	17.57 (8.86, 26.28)	0.021
	*ZNF783* (cg02373627)	7	Gene body	Island	−17.19 (−24.4, −9.98)	0.003
	*MVD* (cg27467516)	16	Gene body	Island	−16.55 (−24.94, −8.16)	0.027
	*TMEM201* (cg07232095)	1	Gene body; 3'UTR		−13.44 (−18.8, −8.08)	0.002
	*CFHR5* (cg13682187)	1	TSS200		11.88 (6.83, 16.94)	0.004

Models were adjusted for bisulfite conversion batch, offspring sex, maternal age, smoking status, occupation and parity.

CHR, chromosome; TSS-1500, 1500 base-pairs from a transcription start site; TSS-200, 200 base-pairs from a transcription start site; UTR, untranslated region. Shores are defined according to NCBI guidelines and labelled north (upstream) or south (downstream) of islands.

*β coefficient is mean difference in percentage methylation compared with offspring of normal weight mothers

### The paternal vs maternal effect of obesity

**Figure 3.**
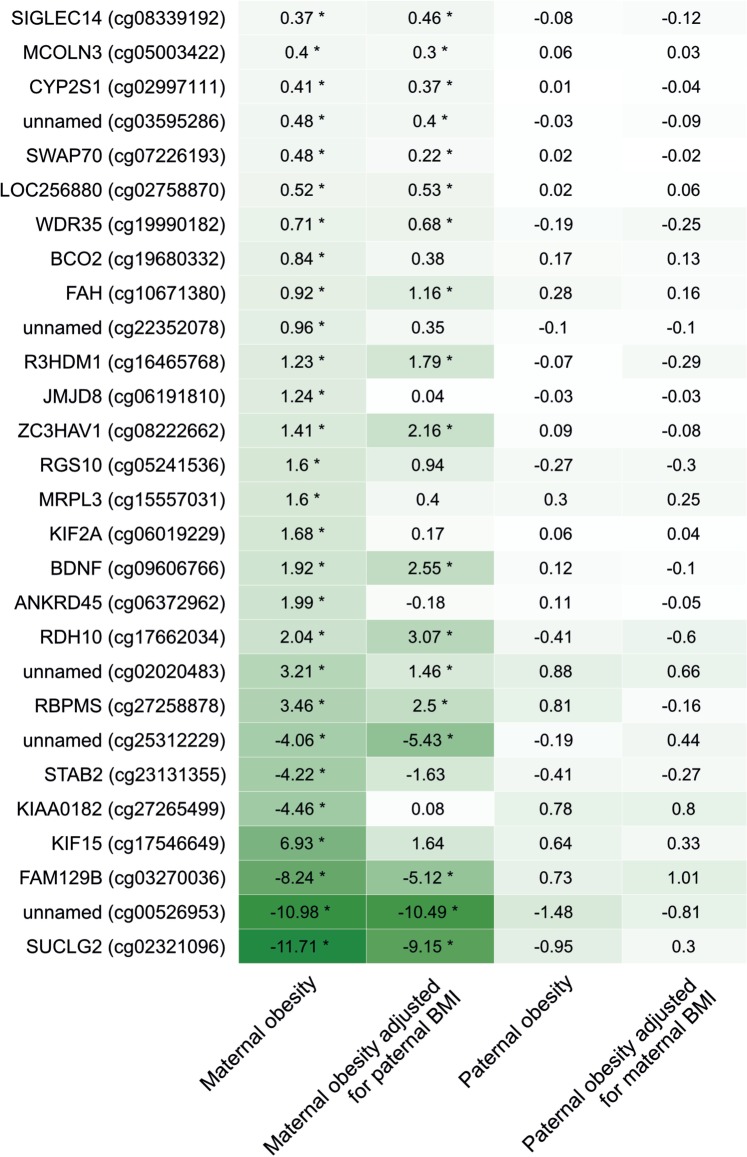
Associations between maternal or paternal obesity and offspring cord blood DNA methylation [mean difference in methylation (%) compared with offspring of normal weight mothers/fathers]. Darker shading indicates a larger effect size (regardless of direction). Models were adjusted for bisulfite conversion batch, and paternal/maternal continuous BMI where indicated, but no other covariates (*n* obese mothers = 40, n normal weight mothers = 665, *n* obese fathers = 53, *n* normal weight fathers = 372). Stars indicate associations with an FDR-adjusted *P*-value < 0.05.

Associations of maternal obesity with offspring cord blood methylation were of greater magnitude than associations of paternal obesity with offspring cord blood methylation at all 28 sites identified through EWAS as associated with maternal obesity (Figure 3). The maternal associations remained stronger than paternal associations after mutual adjustment of maternal and paternal BMI. There was only one underweight father, so we were unable to compare the effect of maternal and paternal underweight.

### Gestational weight gain and offspring methylation at birth

None of our measurements of GWG [total GWG (*n* = 673), GWG in early, mid and late pregnancy (*n* = 690) and IoM-recommended categories of GWG (recommended GWG *n* = 258, under recommended GWG *n* = 242, over recommended GWG *n* = 170)] were associated with cord blood methylation at any site on the array.

### Comparison with previously published results

We quantitatively compared our EWAS results to associations between maternal adiposity and cord blood methylation that have been previously reported in the literature (Table S5, available as Supplementary data at *IJE* online).^21^^,^^22^^,^^23^^,^^24^ Our results agreed with the direction of effect previously reported at one probe at *ZCCHC1* and five probes at *PPARGC1A* in association with maternal pre-pregnancy BMI, one probe at *MMP7* in association with GWG in early pregnancy and 12 probes at *RXRA* in association with maternal pre-pregnancy underweight (assumed to be related to lower maternal carbohydrate intake in early pregnancy, which was the exposure in the previous study^24^). However, with the exception of one probe at *RXRA* [cg14654324, 1.09(0.43, 1.75) difference in percentage methylation in offspring of underweight women compared with normal weight women, *P-*value 0.001], confidence intervals crossed the null and we also found other probes at *PPARGC1A*, *MMP7* and *RXRA* that were associated with maternal adiposity in the reverse direction to that reported previously.

### Persistence of associations at later time-points

Table 2 summarises the results of EWAS (using Model 2) of maternal adiposity and offspring methylation at the three time points—birth, childhood and adolescence. Full results are supplied in File S1 (available as Supplementary data at *IJE* online). There was no overlap between time points in top hits identified, apart from one site (cg23521281 at WDR75), which was positively associated with maternal underweight at birth [0.63 (0.33, 0.93)] and at adolescence [0.77 (0.44, 1.10)]. Of all the exposures, maternal underweight yielded the highest number of hits at each time point.

**Table 2.** The number of CpG sites identified by EWAS as associated with maternal adiposity (FDR-adjusted *P*-value < 0.05) in cord blood at birth, peripheral blood in childhood and peripheral blood in adolescence. Models were adjusted for bisulfite conversion batch, offspring sex, maternal age, smoking status, occupation and parity. Models where GWG was the outcome were additionally adjusted for pre-pregnancy BMI, gestational age at delivery and GWG in the previous stage of pregnancy, where appropriate

EWAS	Neonatal cord blood		Child peripheral blood		Adolescent peripheral blood	
	Number of samples in analysis	Number of CpG sites identified	Number of samples in analysis	Number of CpG sites identified	Number of samples in analysis	Number of CpG sites identified
Maternal pre-pregnancy BMI (continuous)	727	2	770	2	775	0
Maternal obesity	609 (32 obese)	28	646 (26 obese)	2	654 (33 obese)	5
Maternal overweight	671 (94 overweight)	0	710 (98 overweight)	0	716 (95 overweight)	0
Maternal underweight	601 (24 underweight)	1621^a^	638 (26 underweight)	4	647 (26 underweight)	126
GWG in early pregnancy	690	0	733	0	738	0
GWG in mid pregnancy	690	0	733	0	738	0
GWG in late pregnancy	690	0	733	0	738	0
Total GWG (over entire pregnancy)	673	0	712	0	715	0
Below IOM-recommended GWG	500 (242 under recommended GWG)	0	530 (258 under recommended GWG)	0	538 (260 under recommended GWG)	0
Over IOM-recommended GWG	428 (170 over recommended GWG)	0	454 (182 over recommended GWG)	0	454 (176 over recommended GWG)	0

^a^1 site is common to birth and adolescence.

**Figure 4.**
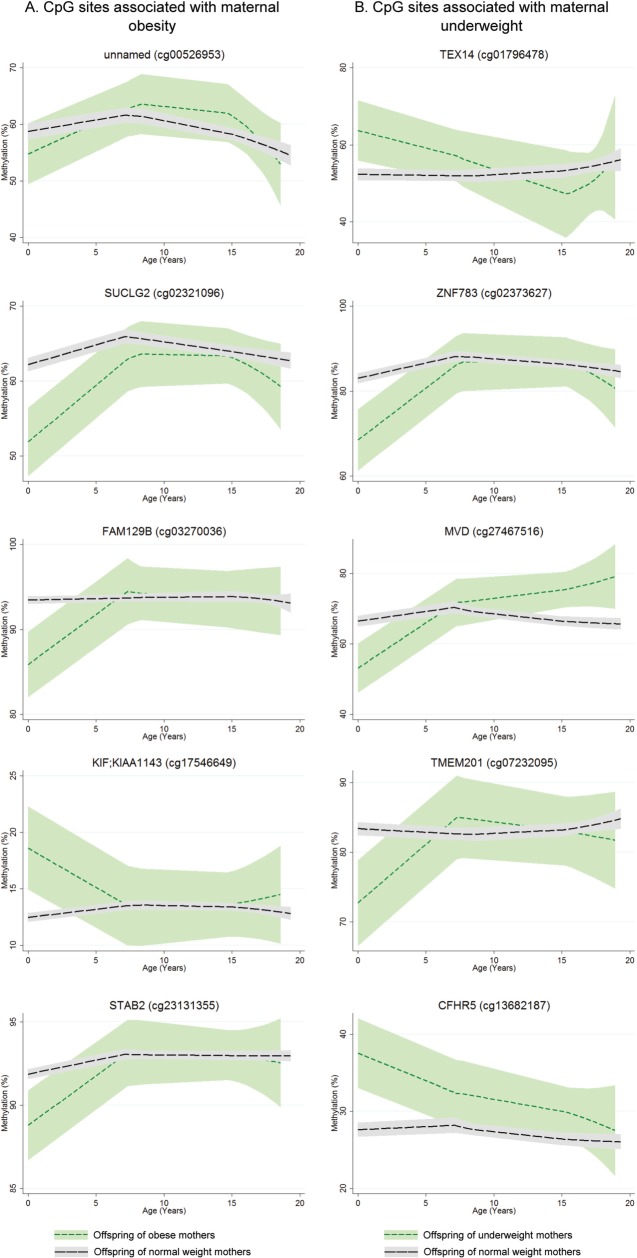
Methylation (%) over time for offspring of obese (short dashed (green) line in a) or underweight (short dashed (green) line in b) mothers compared with offspring of normal weight mothers (long dashed (black) line). Ribbons indicate 95% confidence intervals.

Explicit longitudinal modelling of methylation at sites identified in cord blood as being associated with maternal obesity or underweight showed that this lack of overlap in EWAS hits between time points is likely due to postnatal resolution of differential methylation. Full results of this longitudinal analysis are presented in File S2 (available as Supplementary data at *IJE* online). Table 3 and Figure 4a show results of longitudinal analysis of the top five sites found differentially methylated in cord blood, between offspring of normal weight and obese mothers. At these sites, changes in methylation were stronger in early/mid childhood (age 0 to 7 years) than in late childhood/adolescence (age 7 to 17 years). Differences in methylation change were found at three of the top five sites. For example, at cg03270036 (on a CpG island shore at FAM129B), the estimated cord blood methylation in offspring of obese mothers was 8.9% lower than in offspring of normal weight mothers. During early/mid childhood, methylation decreased in the offspring of normal weight mothers but increased in the offspring of obese mothers (*P*-value for difference in changes during childhood 4 × 10^−9^), i.e. methylation differences here were being resolved during early childhood. During late childhood/adolescence however, offspring from both BMI groups had decreasing methylation, with no difference in rate of change. The effect of cellular heterogeneity from cord to peripheral blood is strong at cg00526953 (on a CpG island shore at an uncharacterized locus on chromosome 16): in Figure 4a, methylation increases during childhood for offspring of both BMI groups, whereas in Table 3 methylation decreases with age. This suggests that changing cell-type proportions are associated with increasing methylation, and after controlling for this, methylation decreases with age on average.

**Table 3.** Longitudinal analysis of top five sites found differentially methylated in cord blood, between offspring of normal weight and obese mothers

Gene region (CpG)	Estimated cord blood methylation in offspring of normal weight mothers (%)	Estimated cord blood methylation in offspring of obese mothers (%)	Age 0–7 years: Average yearly change for offspring of normal weight mothers (%)	Age 0–7 years: Average yearly change for offspring of obese mothers (%)	Age 0–7 years: *P*-value for difference in methylation change	Age 7–17 years: Average yearly change for offspring of normal weight mothers (%)	Age 7–17 years: Average yearly change for offspring of obese mothers (%)	Age 7–17 years: *P*-value for difference in methylation change
unnamed (cg00526953)	70.33	66.17	−2.00	−1.27	0.13	−0.49	−0.39	0.76
*SUCLG2* (cg02321096)	61.60	54.52	0.96	1.84	0.007	−0.09	−0.13	0.88
*FAM129B* (cg03270036)	96.92	88.02	−0.66	0.77	4.13E-09	−0.14	−0.32	0.28
*KIF15;KIAA1143* (cg17546649)	9.92	14.97	0.46	−0.20	0.0003	0.01	0.00	0.90
*STAB2* (cg23131355)	92.73	88.21	0.10	0.68	1.29E-05	−0.07	−0.07	0.96

Table 4 and Figure 4b show results of longitudinal analysis of the top five sites found differentially methylated in cord blood, between offspring of normal weight and underweight mothers. At these sites, changes in methylation again acted to resolve differences found in cord blood. For example, at cg07232095 (on the gene body of TMEM201), cord blood methylation was almost 12% higher in the offspring of normal weight mothers. During childhood however, there was almost no change for these children, whereas methylation in the offspring of underweight mothers increased on average by 1.75% per year.

**Table 4.** Longitudinal analysis of top five sites found differentially methylated in cord blood, between offspring of normal weight and underweight mothers

Gene region (CpG)	Estimated cord blood methylation in offspring of normal weight mothers (%)	Estimated cord blood methylation in offspring of underweight mothers (%)	Age 0–7 years: Average yearly change for offspring of normal weight mothers (%)	Age 0–7 years: Average yearly change for offspring of underweight mothers (%)	Age 0–7 years: *P*-value for difference in methylation change	Age 7–17 years: Average yearly change for offspring of normal weight mothers (%)	Age 7–17 years: Average yearly change for offspring of underweight mothers (%)	Age 7–17 years: *P*-value for difference in methylation change
*TEX14* (cg01796478)	52.95	63.76	1.03	−0.19	0.09	−0.28	−0.60	0.51
*ZNF783* (cg02373627)	89.94	73.92	−1.02	1.46	1.94E-05	−0.12	−0.43	0.44
*MVD* (cg27467516)	68.14	56.90	0.03	1.75	0.02	0.11	1.05	0.07
*TMEM201* (cg07232095)	83.72	71.95	0.00	1.75	3.23E-05	−0.11	−0.10	0.97
*CFHR5* (cg13682187)	25.21	36.63	0.61	−0.23	0.01	−0.34	−0.74	0.09

### Methylation at birth and adiposity in childhood and adolescence

**Figure 5.**
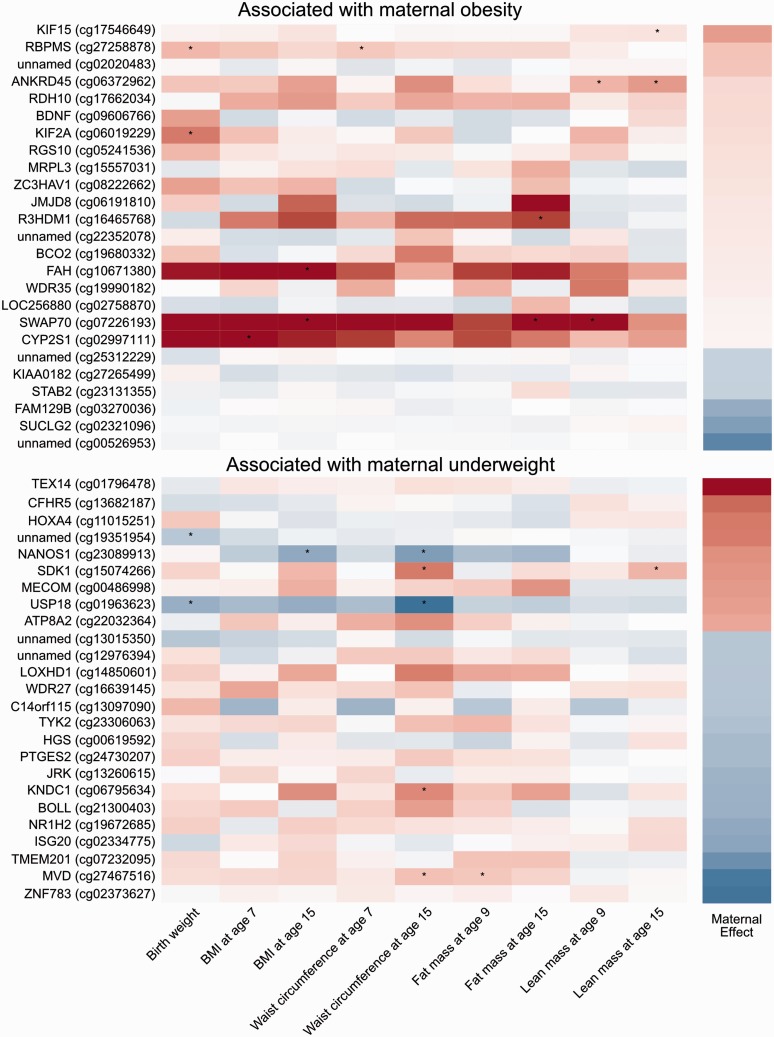
Associations between cord blood DNA methylation and offspring adiposity. The heatmaps are built using effect sizes. Positive associations appear more red and negative associations appear more blue. CpG sites were selected based on their association with maternal underweight or obesity, the effect size for which is indicated in the far right-hand panel. Stars indicate associations with a *P*-value < 0.05 (before correction for multiple testing).

Figure 5 shows effect sizes for associations between methylation at birth and various measures of offspring adiposity (results are shown for the top 25 CpG sites with the largest effect sizes and an FDR-adjusted *P*-value < 0.05 for the association between maternal underweight/obesity and cord blood methylation). In general, sites that were hypermethylated in association with maternal obesity or hypomethylated in association with maternal underweight tended to be positively associated with offspring adiposity, and sites hypomethylated in association with maternal obesity or hypermethylated in association with maternal underweight tended to be inversely associated with offspring adiposity. This trend is strongest for birthweight and largely consistent over different measures of offspring adiposity recorded at different times (Table S6, available as Supplementary data at *IJE* online). It should be noted that associations between offspring adiposity and cord blood methylation at individual sites did not survive correction for multiple testing.

## Discussion

In support of a previously postulated effect of maternal adiposity on the offspring epigenome,^11^^,^^12^^,^^13^ we found several differentially methylated CpG sites in cord blood of offspring of obese and underweight mothers compared with offspring of normal weight mothers, with no overlap in the sites that maternal obesity and underweight relate to. Despite being less powered to detect associations, the comparison of underweight with normal weight mothers identified many more differentially methylated sites (1621) than the comparison of obese with normal weight women (28). However, we found no evidence of genomic inflation in either analysis, suggesting that maternal underweight has a larger effect on the fetal epigenome than maternal obesity does, though we acknowledge the need to replicate our findings. We did not find any differentially methylated CpG sites when comparing less extreme groups (i.e. offspring of overweight mothers compared with offspring of normal weight mothers) or when assessing methylation in relation to maternal pre-pregnancy BMI over the whole distribution (i.e. when BMI was analysed as a continuous variable). This has implications for future studies of maternal pre-pregnancy BMI and offspring DNA methylation, which may benefit from taking a similar approach by comparing DNA methylation between WHO BMI categories.

Using paternal obesity as a negative control, we found that for all of the top CpG sites associated with maternal obesity, the effect on DNA methylation of maternal obesity was stronger than the effect of paternal obesity, even after adjustment for paternal BMI. This suggests that the associations of maternal obesity with offspring methylation at birth are likely to be causally driven via an intrauterine mechanism. In line with these findings, we also noted stronger associations of maternal obesity with offspring adiposity than equivalent paternal-offspring associations. Previous studies in ALSPAC that were not restricted only to those with DNA methylation data have shown that across the continuum, greater unit maternal BMI is on average slightly more strongly associated with offspring fat mass than is paternal BMI, but that study did not look at BMI categories.^38^ There were too few underweight fathers to compare the effects of paternal underweight with maternal underweight on offspring methylation.

We also investigated offspring methylation at birth in relation to total GWG, GWG in early, mid and late pregnancy and IOM categories of recommended GWG, but found no CpG sites associated with variation in any of these measures. This might suggest that the associations of GWG with offspring adiposity that have been reported previously in ALSPAC^2^ and other cohorts^42^^–^^44^ are not mediated by DNA methylation measured in cord blood or whole blood. However, it should be noted that in the ARIES subset of ALSPAC, we did not find strong associations of GWG with offspring adiposity (though these have been found in the whole cohort^2^). Additionally, according to IoM criteria for recommended GWG, relatively few (25%) of the women in our study had excessive GWG. Further exploration in other cohorts with similarly detailed information on GWG will be necessary before any firm conclusions can be drawn. Associations between maternal obesity or maternal underweight and methylation at birth did not consistently persist to other time points. Compared with offspring of normal weight mothers, offspring of mothers who are obese or underweight show differential methylation at several CpG sites at birth, childhood and adolescence, but very few sites were common to more than one time point. Longitudinal analysis revealed that methylation differences between offspring of obese/underweight mothers and normal weight mothers observed at birth were being resolved in early childhood. This could suggest that the association between maternal adiposity and child/adolescent DNA methylation is confounded or mediated by other factors such as genetic inheritance of adiposity, learnt eating behaviours, physical activity, stress responses or other factors arising from a shared environment. Although associations between maternal adiposity and DNA methylation at birth do not appear to persist in later life, it is also possible that variation in the epigenome at birth might have a phenotypic effect that is not limited to the neonatal period, but that triggers physiological pathways that have an apparent impact on the phenotype only in later life.

In contrast to previous observational evidence of a U-shaped relationship between maternal adiposity/nutrition and offspring adiposity, where extreme obesity/overnutrition or extreme underweight/undernutrition are both associated with greater offspring adiposity,^1^^,^^3^^,^^6–8^^,^^45^ maternal underweight was associated with lower offspring adiposity and maternal obesity was associated with greater offspring adiposity in the ARIES cohort. A possible explanation is that, unlike these previous studies, ARIES does not contain individuals at the far extremes of the BMI distributions, such as those that might be undergoing bariatric surgery or experiencing famine.

Although we identified associations of maternal underweight and obesity with methylation in a number of CpG sites in offspring cord blood, few robust associations between methylation at these sites and later offspring adiposity were found. However, there was a general trend showing that sites that were hypermethylated in association with maternal obesity or hypomethylated in association with maternal underweight tended to be positively associated with offspring adiposity, and sites hypomethylated in association with maternal obesity or hypermethylated in association with maternal underweight tended to be inversely associated with offspring adiposity. This suggests that a linear association (as seen in ALSPAC) between maternal adiposity and offspring adiposity might be mediated via offspring methylation at birth. However, further research using larger cohorts and causal analysis techniques such as two-step Mendelian randomization^27^^,^^46^ will be necessary before confounding can be ruled out. We did not apply Mendelian randomization in this study because associations between offspring methylation and adiposity were weak. As with all areas of research, epigenetic epidemiology greatly benefits from attempts to replicate results in different cohorts. Although we did not conduct a formal replication analysis in this study, we did quantitatively compare our results with those previously reported in the literature (Table S5, available as Supplementary data at *IJE* online).^21^^–^^24^ Our analyses did not consistently replicate the direction or magnitude of all previously reported associations between maternal adiposity/nutrition and CpG site methylation, but we did find the same direction of association at several sites. These included several probes on *RXRA* where we found that maternal underweight was associated with greater cord blood methylation. This is in agreement with Godfrey *et al*.^24^ who found that low maternal carbohydrate intake (in the context of famine) was associated with greater methylation at this locus. However, we also found sites at RXRA where maternal underweight was associated with lower methylation. Possible reasons for discrepancies between our results include the fact the mothers included in ALSPAC as a whole and those selected to ARIES are mostly normal or overweight and it is possible that additional associations would have been seen comparing groups with more extreme BMI levels. Stringent statistical analysis provides confidence in our observations, although further work involving meta-analysis of EWAS results from multiple cohorts and extensive interrogation of potential causal relationships is warranted.

Limitations of our study include the limited coverage of the HumanMethylation 450 K array—although the array provides a relatively inexpensive, high-throughput solution to epigenome-wide profiling, it only covers around 1.7% of all CpG sites in the genome.^47^ Our study relies on data from cord or peripheral blood leukocytes, which may not be the most appropriate tissue in which to study associations with adiposity. Such associations may be tissue-specific, so future work should focus on more in-depth analysis of identified loci, possibly using targeted bisulfite sequencing, in adipose tissue, which is more relevant to the development of adiposity and may have distinct DNA methylation signatures.^48^ Further work to relate changes in DNA methylation to gene and protein expression will also be necessary to fully elucidate the mechanisms at play. It is also possible that pre-pregnancy BMI and GWG alone are insufficient as markers for over/under-nutrition or subsequent metabolic problems. This might explain the absence of widespread associations of offspring methylation with maternal BMI or GWG within women closer to the healthy range. Measurement error in BMI, in particular the possibility of systematic error in tself-reported weight, might have biased our findings. However, our comparisons of this self-report with predicted pre-pregnancy weight based on all repeat antenatal clinic measures in mothers and with measured postnatal weight in fathers suggested little evidence of systematic mis-reporting. Finally, in our comparison of associations between maternal vs paternal obesity and offspring DNA methylation, we did not formally account for the possibility of non-paternity,^37^^,^^49^ although we did exclude partners who were not the biological father of the offspring according to the mother’s report.

In conclusion, we have identified several CpG sites that are differentially methylated in cord blood of offspring of obese or underweight mothers compared with offspring of normal weight mothers and our results suggest an intrauterine mechanism. Our results suggest that maternal underweight might be more influential on offspring DNA methylation than maternal obesity and weight gain during pregnancy appears to have little effect. We found some evidence that associations of maternal adiposity with offspring adiposity may be mediated via DNA methylation in blood. Our findings should be treated with some caution until they have been replicated in large independent studies and further exploration of causality with methods, such as two-step Mendelian randomization, in collaborations with adequate statistical power have been completed.

## Supplementary Data

Supplementary data are available at *IJE* online.

## Funding

This work was supported by the MRC Integrative Epidemiology Unit and the University of Bristol (MC_UU_12013_2, MC_UU_12013_5 and MC_UU_12013_8) and the United States National Institute of Diabetes and Digestive and Kidney Diseases (R01 DK10324). A.F. is funded by a UK Medical Research Council research fellowship (0701594/1). R.C.R. is funded by a Wellcome Trust 4-year PhD studentship (Grant Code: WT083431MF). G.D.S. and C.L.R. are partially supported by the ESRC (RES-060-23-0011) ‘The biosocial archive: transforming lifecourse social research through the incorporation of epigenetic measures’. G.D.S.’s work is supported in part by the European Research Council grant DEVHEALTH 269874. D.A.L.’s work on this paper is in part supported by the Wellcome Trust (WT094529MA). The UK Medical Research Council and the Wellcome Trust (Grant ref: 102215/2/13/2) and the University of Bristol provide core support for ALSPAC. The Accessible Resource for Integrated Epigenomics Studies (ARIES) was funded by the UK Biotechnology and Biological Sciences Research Council (BB/I025751/1 and BB/I025263/1). GWAS data were generated by Sample Logistics and Genotyping Facilities at the Wellcome Trust Sanger Institute and LabCorp (Laboratory Corporation of America) using support from 23andMe. The funders had no role in study design, data collection and analysis, decision to publish, or preparation of the manuscript. This publication is the work of the authors and G.C.S. will serve as a guarantor for the contents of this paper.

## Supplementary Material

Supplementary Data

## References

[1] Lumey LH Stein AD Susser E Prenatal famine and adult health. Ann Rev Public Halth 2011;32:237–62. 10.1146/annurev-publhealth-031210-101230 PMC3857581 21219171

[2] Fraser A Tilling K Macdonald-Wallis C Association of maternal weight gain in pregnancy with offspring obesity and metabolic and vascular traits in childhood. Circulation 2010;121:2557–64. 20516377 10.1161/CIRCULATIONAHA.109.906081 PMC3505019

[3] Lawlor DA Relton C Sattar N Nelson SM Maternal adiposity - a determinant of perinatal and offspring outcomes? Nat Rev Endocrinol 2012;8:679–88. 23007319 10.1038/nrendo.2012.176

[4] Lawlor DA The Society for Social Medicine John Pemberton Lecture 2011. Developmental overnutrition—an old hypothesis with new importance? Int J Epidemiol 2013;42:7–29. 23508404 10.1093/ije/dys209

[5] Lawlor DA Lichtenstein P Fraser A Långström N Does maternal weight gain in pregnancy have long-term effects on offspring adiposity? A sibling study in a prospective cohort of 146,894 men from 136,050 families. Am J Clin Nutr 2011;94:142–48. 21562086 10.3945/ajcn.110.009324 PMC3127508

[6] Lawlor DA Lichtenstein P Långström N Association of maternal diabetes mellitus in pregnancy with offspring adiposity into early adulthood: sibling study in a prospective cohort of 280,866 men from 248,293 families. Circulation 2011;123:258–65. 21220735 10.1161/CIRCULATIONAHA.110.980169 PMC4440894

[7] Kral JG Biron S Simard S Large maternal weight loss from obesity surgery prevents transmission of obesity to children who were followed for 2 to 18 years. Pediatrics 2006;118:e1644–49. 17142494 10.1542/peds.2006-1379

[8] Smith J Cianflone K Biron S Effects of maternal surgical weight loss in mothers on intergenerational transmission of obesity. *J Clin Endocrinol Metab*. 2009;94:4275–83. 19820018 10.1210/jc.2009-0709

[9] Berglind D Willmer M Näslund E Tynelius P Sørensen TIA Rasmussen F Differences in gestational weight gain between pregnancies before and after maternal bariatric surgery correlate with differences in birth weight but not with scores on the body mass index in early childhood. Pediatr Obes 2014;9:427–34 24339139 10.1111/j.2047-6310.2013.00205.x

[10] Willmer M Berglind D Sørensen TIA Näslund E Tynelius P Rasmussen F Surgically induced interpregnancy weight loss and prevalence of overweight and obesity in offspring. PloS One 2013;8(:e82247. 24349234 10.1371/journal.pone.0082247 PMC3861408

[11] Lillycrop KA Burdge GC Epigenetic changes in early life and future risk of obesity. Int J Obes (Lond) 2011;35:72–83. 20548303 10.1038/ijo.2010.122

[12] Mathers JC Early nutrition: impact on epigenetics. Forum Nutr 2007;60:42–48. 17684400 10.1159/000107066

[13] Li CCY Maloney CA Cropley JE Suter CM Epigenetic programming by maternal nutrition: shaping future generations. Epigenomics 2010;2:539–49. 22121973 10.2217/epi.10.33

[14] Heijmans BT Tobi EW Stein AD Persistent epigenetic differences associated with prenatal exposure to famine in humans. Proc Natl Acad Sci U S A 2008;105:17046–49. 18955703 10.1073/pnas.0806560105 PMC2579375

[15] Tobi EW Lumey LH Talens RP DNA methylation differences after exposure to prenatal famine are common and timing- and sex-specific. Hum Mol Genet 2009;18:4046–53. 19656776 10.1093/hmg/ddp353 PMC2758137

[16] Waterland RA Kellermayer R Laritsky E Season of conception in rural Gambia affects DNA methylation at putative human metastable epialleles. PLoS Genet 2010;6:e1001252. 21203497 10.1371/journal.pgen.1001252 PMC3009670

[17] Dominguez-Salas P Moore SE Baker MS Maternal nutrition at conception modulates DNA methylation of human metastable epialleles. Nat Communications 2014;5:3746. 10.1038/ncomms4746 PMC4015319 24781383

[18] Guénard F Tchernof A Deshaies Y Methylation and expression of immune and inflammatory genes in the offspring of bariatric bypass surgery patients. J Obes 2013;2013:492170. 23840945 10.1155/2013/492170 PMC3693160

[19] Michels KB Harris HR Barault L Birthweight, maternal weight trajectories and global DNA methylation of LINE-1 repetitive elements. PloS One 2011;6:e25254. 21980406 10.1371/journal.pone.0025254 PMC3182185

[20] Herbstman JB Wang S Perera FP Predictors and consequences of global DNA methylation in cord blood and at three years. PloS One 2013;8:e72824. 24023780 10.1371/journal.pone.0072824 PMC3762861

[21] Morales E Groom A Lawlor DA Relton CL DNA methylation signatures in cord blood associated with maternal gestational weight gain: results from the ALSPAC cohort. BMC Res Notes 2014;7:278. 24886386 10.1186/1756-0500-7-278 PMC4108052

[22] Liu X Chen Q Tsai H-J Maternal preconception body mass index and offspring cord blood DNA methylation: exploration of early life origins of disease. Environ Mol Mutagen 2014;55:223–30. 24243566 10.1002/em.21827 PMC4547934

[23] Gemma C Sookoian S Alvariñas J Maternal pregestational BMI is associated with methylation of the PPARGC1A promoter in newborns. Obesity (Silver Spring) 2009;17:1032–39. 19148128 10.1038/oby.2008.605

[24] Godfrey KM Sheppard A Gluckman PD Epigenetic gene promoter methylation at birth is associated with child’s later adiposity. Diabetes 2011;60:1528–34. 21471513 10.2337/db10-0979 PMC3115550

[25] Relton CL Groom A St Pourcain B DNA methylation patterns in cord blood DNA and body size in childhood. PloS One 2012;7:e31821. 22431966 10.1371/journal.pone.0031821 PMC3303769

[26] Davey Smith G Negative control exposures in epidemiologic studies. Epidemiology 2012;23:350–51; author reply 351–52. 22317815 10.1097/EDE.0b013e318245912c

[27] Richmond R Al-Amin A Davey Smith G Relton C Approaches for drawing causal inferences from epidemiological birth cohorts: A review. *Early Human Development*. 2014;90:769–80. 25260961 10.1016/j.earlhumdev.2014.08.023 PMC5154380

[28] Boyd A Golding J Macleod J Cohort Profile: The ‘children of the 90s'—the index offspring of the Avon Longitudinal Study of Parents and Children. Int J Epidemiol 2013;42:111–27. 22507743 10.1093/ije/dys064 PMC3600618

[29] Fraser A Macdonald-Wallis C Tilling K Cohort Profile: The Avon Longitudinal Study of Parents and Children: ALSPAC mothers cohort. Int J Epidemiol 2013;42:97–110. 22507742 10.1093/ije/dys066 PMC3600619

[30] Macdonald-Wallis C Lawlor DA Palmer T Tilling K Multivariate multilevel spline models for parallel growth processes: application to weight and mean arterial pressure in pregnancy. Stat Med 2012;31:3147–64. 22733701 10.1002/sim.5385 PMC3569877

[31] Institute of Medicine (US) and National Research Council (US) Committee to Reexamine IOM Pregnancy Weight Guidelines. Rasmussen K Yaktine A (eds). Weight Gain During Pregnancy: Reexamining the Guidelines. Washington, DC: National Academies Press, 2009. 20669500

[32] Bibikova M Barnes B Tsan C High density DNA methylation array with single CpG site resolution. Genomics 2011;98:288–95. 21839163 10.1016/j.ygeno.2011.07.007

[33] Schalkwyk LC Pidsley R Wong CC wateRmelon: Illumina 450 methylation array normalization and metrics. R package version 1.5.1. 2013.

[34] Touleimat N Tost J Complete pipeline for Infinium(®) Human Methylation 450K BeadChip data processing using subset quantile normalization for accurate DNA methylation estimation. Epigenomics 2012;4:325–41. 22690668 10.2217/epi.12.21

[35] Aryee MJ Jaffe AE Corrada-Bravo H Minfi: a flexible and comprehensive Bioconductor package for the analysis of Infinium DNA methylation microarrays. Bioinformatics 2014;30:1363–69. 24478339 10.1093/bioinformatics/btu049 PMC4016708

[36] Houseman EA Accomando WP Koestler DC DNA methylation arrays as surrogate measures of cell mixture distribution. BMC Bioinformatics 2012;13:86. 22568884 10.1186/1471-2105-13-86 PMC3532182

[37] Davey Smith G Steer C Leary S Ness A Is there an intrauterine influence on obesity? Evidence from parent child associations in the Avon Longitudinal Study of Parents and Children (ALSPAC). Arch Dis Child 2007;92:876–80. 17595200 10.1136/adc.2006.104869 PMC2083247

[38] Lawlor DA Timpson NJ Harbord RM Exploring the developmental overnutrition hypothesis using parental-offspring associations and FTO as an instrumental variable. PLoS Med 2008;5:e33. 18336062 10.1371/journal.pmed.0050033 PMC2265763

[39] Du P Zhang X Huang C-C Comparison of Beta-value and M-value methods for quantifying methylation levels by microarray analysis. BMC Bioinformatics 2010;11:587. 21118553 10.1186/1471-2105-11-587 PMC3012676

[40] Laird N Ware J Random-effects for longitudinal data. Biometrics 1982;3:963–974. 7168798

[41] Goldstein H Multilevel mixed linear model analysis using iterative generalized least squares. Biometrika 1986;73:43–56.

[42] Wrotniak BH Shults J Butts S Stettler N Gestational weight gain and risk of overweight in the offspring at age 7 y in a multicenter, multiethnic cohort study. Am J Clin Nutr 2008;87:1818–24. 18541573 10.1093/ajcn/87.6.1818

[43] Oken E Taveras EM Kleinman KP Rich-Edwards JW Gillman MW Gestational weight gain and child adiposity at age 3 years. Am J Obstet Gynecol 2007;196:322 e1–8. 17403405 10.1016/j.ajog.2006.11.027 PMC1899090

[44] Moreira P Padez C Mourão-Carvalhal I Rosado V Maternal weight gain during pregnancy and overweight in Portuguese children. Int J Obes (Lond) 2007;31:608–14. 17384661 10.1038/sj.ijo.0803582

[45] Lawlor DA Fraser A Macdonald-Wallis C Maternal and offspring adiposity-related genetic variants and gestational weight gain. Am J Clin Nutr 2011;94:149–55. 21593506 10.3945/ajcn.110.010751 PMC3127507

[46] Relton CL Davey Smith G Two-step epigenetic Mendelian randomization: a strategy for establishing the causal role of epigenetic processes in pathways to disease. Int J Epidemiol 2012;41:161–76. 22422451 10.1093/ije/dyr233 PMC3304531

[47] Clark C Palta P Joyce CJ A comparison of the whole genome approach of MeDIP-seq to the targeted approach of the Infinium HumanMethylation450 BeadChip(®) for methylome profiling. PloS One 2012;7:e50233. 23209683 10.1371/journal.pone.0050233 PMC3510246

[48] Slieker RC Bos SD Goeman JJ Identification and systematic annotation of tissue-specific differentially methylated regions using the Illumina 450k array. Epigenet Chromatin 2013;6:26. 10.1186/1756-8935-6-26 PMC3750594 23919675

[49] Patel R Martin RM Kramer MS Familial associations of adiposity: findings from a cross-sectional study of 12,181 parental-offspring trios from Belarus. PloS One 2011;6(1):e14607. 21298034 10.1371/journal.pone.0014607 PMC3029263

