# Supplementary material for: Maternal pre-pregnancy BMI and gestational weight gain, offspring DNA methylation and later offspring adiposity: findings from the Avon Longitudinal Study of Parents and Children
Source: Int J Epidemiol. 2015 Apr 8;44(4):1288–304. doi: 10.1093/ije/dyv042 (PMC4588865; doi:10.1093/ije/dyv042)
Supplement: Supplementary Data [file supp_44_4_1288__index.html]

Maternal pre-pregnancy BMI and gestational weight gain, offspring DNA methylation and later offspring adiposity: findings from the Avon Longitudinal Study of Parents and Children — Supplementary Data 

# Maternal pre-pregnancy BMI and gestational weight gain, offspring DNA methylation and later offspring adiposity: findings from the Avon Longitudinal Study of Parents and Children

## Supplementary Data

files

**Files in this Data Supplement:**

- Supplementary Data - pdf file
- Supplementary Data - xls file
- Supplementary Data - xlsx file
