# Supplementary material for: Maternal pre-pregnancy BMI and gestational weight gain, offspring DNA methylation and later offspring adiposity: findings from the Avon Longitudinal Study of Parents and Children
Source: Int J Epidemiol. 2015 Apr 8;44(4):1288–304. doi: 10.1093/ije/dyv042 (PMC4588865; doi:10.1093/ije/dyv042)
Supplement: Supplementary Data [file supp_dyv042_Supplementary_text_figures_and_tables.pdf]

Supplementary online material

Bland Altman Plots to explore systematic bias in reported weight

In order to explore the possibility that systematic bias in partner or self-report weight (e.g. those who are heavier systematically under-reporting their weight) might bias our findings we used Bland-Altman plots (plots of mean and difference in mean).

Maternal self-reported pre-pregnancy weight with estimated pre-pregnancy weight (estimated from multi-level models using all measured weights during pregnancy). On average self-reported pre-pregnancy weight tended to be slightly higher than pre-pregnancy weight estimated from the antenatal clinic measurements, with the mean of predicted from clinic measures minus self-report being -1.4kg (95% CI:-1.5, -1.3) and 95% limits of agreement of -9.5 to -6.7kg. The Bland-Altman plot (below) suggests that the level of misreporting is similar for the majority of participants and is not markedly influenced by mean weight, with a weak positive correlation between the mean and difference of the two measurements (r = 0.16).

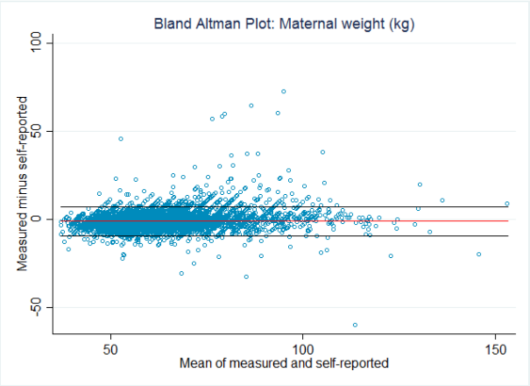

Paternal partner-reported weight with measured weight 21 years later. Although paternal weight around the time of pregnancy was not measured, we found that partner-reported paternal weight at this time tended to be lower than that measured 21 years later, with the mean of measured minus partner-report being 9.8 (95%CI: 9.3, 10.2) and 95% limits of agreement of -8.9 to 28.5. The Bland-Altman plot (below) suggests that the level of misreporting is similar for the majority of participants and is not markedly influenced by mean weight, with a weak positive correlation between the mean and difference of the two measurements (r=0.40).

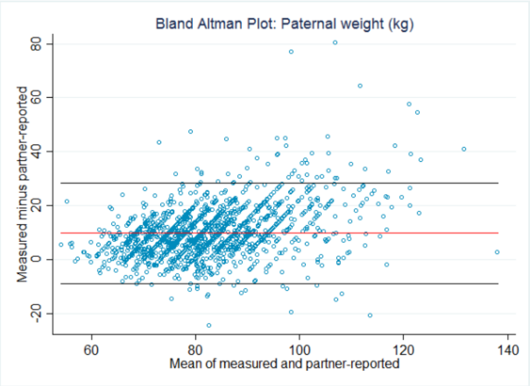

Details of the collection and generation of DNA methylation data

Cord or peripheral blood (whole blood or buffy coats) were collected according to standard procedures, spun and frozen at -80˚C. DNA methylation analysis and data pre-processing were performed at the University of Bristol as part of the ARIES project (ariesepigenomics.org.uk). Following extraction, DNA was bisulfite converted using the Zymo EZ DNA MethylationTM kit (Zymo, Irvine, CA). Following conversion, the genome-wide methylation status of over 485,000 CpG sites was measured using the Illumina Infinium® HumanMethylation450k BeadChip assay according to the standard protocol. The arrays were scanned using an Illumina iScan and initial quality review was assessed using GenomeStudio (version 2011.1). The level of methylation is expressed as a "Beta" value (β-value), ranging from 0 (no cytosine methylation) to 1 (complete cytosine methylation). Samples from all time-points in ARIES were distributed across slides using a semi-random approach (sampling criteria were in place to ensure that all time-points were represented on each array) to minimize the possibility of confounding by batch effects. In addition, during the data generation process a wide range of batch variables were recorded in a purpose-built laboratory information management system (LIMS). The LIMS also reported QC metrics from the standard control probes on the HumanMethylation450k BeadChip for each sample back to the laboratory. Of all measured batch variables, bisulfite conversion batch (96-well plate) was identified as by far the most influential on the ARIES HumanMethylation450k data (Figure S1), Slide level batch adjustment is less useful as each slide will only contain a small number of samples for each time point, additionally allocation to bisulfite conversion batch is more likely to contain systematic bias because samples were added to the batch according to lab priorities and convenience. Samples failing quality control (average probe detection p-value ≥ 0.01) were repeated. As an additional quality control step genotype probes on the HumanMethylation450k were compared between samples from the same individual and against SNP-chip data to identify and remove any sample mismatches.

Data were pre-processed in R (version 3.0.1) with the WateRmelon package[1] according to the subset quantile normalization approach described by Touleimat & Tost[2] in an attempt to reduce the non-biological differences between probes. Sites on sex chromosomes were excluded to reduce complexity due to sex-specific differences and X-chromosome inactivation by DNA methylation in females. We excluded probes identified by Naeem *et al.*[3] that map to multiple genomic locations, contain known repeat regions, contain known INDELs, contain SNPs, or are affected by other unknown/multiple factors. Finally, we also excluded probes showing a detection P-value >0.05 for >5% samples. This left 284 972, 285 929 and 285 656 probes for analysis in neonatal cord blood, peripheral blood in childhood and peripheral blood in adolescence, respectively.

EWAS regression model optimisation

The U-shaped association between maternal and offspring adiposity (where adiposity is greater in offspring of underweight or obese mothers) led us to hypothesise that any association between maternal adiposity and offspring adiposity might also be non-linear, so we tested the assumption of linear relationships between GWG/pre-pregnancy BMI and offspring methylation by performing linear regression using the top 1000 most variable probes, firstly with the exposure untransformed and secondly with a quadratic term for the exposure. We used a likelihood ratio test to interpret whether or not the model fit was improved by the inclusion of the quadratic term. However, likelihood ratio tests showed that the assumption of linear relationships between pre-pregnancy BMI/GWG and cord blood methylation is valid (for 93.6%-94.9% of probes the model fit was not improved (likelihood ratio test P-value >0.05) by the inclusion of a quadratic term for pre-pregnancy BMI/GWG).

We also tested the hypothesis that there is an interaction between continuous pre-pregnancy BMI and continuous stage-specific or total GWG that should be considered in model design. Again, linear regression was performed on the top 1000 most variable probes, firstly with no interaction and secondly with an interaction (stage-specific or total GWG x pre-pregnancy BMI). Likelihood ratio tests were used to assess whether or not the model fit was improved by the inclusion of the interaction. Likelihood ratio tests also showed that for models where GWG is the exposure, model fit was not improved by including pre-pregnancy BMI as an interaction with GWG rather than as a confounder (the P-value was >0.05 for 89.0 to 94.2% of 1000 probes tested).

It has been suggested that logit-transforming β-values to "M-values" gives a better approximation of a normal distribution and is therefore more statistically valid. However, interpretation of β-values (on a scale of 0 (completely unmethylated) to 1 (completely methylated)) is more intuitive.[4] We performed linear regression of methylation as β-values or M-values on pre-pregnancy BMI using the top 1000 most variable probes. A similar number of differentially methylated CpG sites (P-value <0.05) were identified using M-values (51) and β-values (56). Of these, 52 sites were identified using both methods (67.2% agreement). Therefore, we consider that β-values are appropriate for our analyses.

Longitudinal model

Longitudinal methylation data were extracted for each of these CpG sites. A multilevel model[1,2] including a random intercept and a linear regression spline term to allow for flexibility was fitted to each of these sites sequentially. For example, for sites found when comparing obese and normal weight mothers:

$$meth_{ij} = \beta_0 + u_{0i} + \beta_1 Obese_i + \beta_2 age_{ij} + \beta_3 (age_{ij} - 7)_+ + \beta_4 Obese_i age_{ij} + \beta_5 Obese_i (age_{ij} - 7)_+ + confounders + \varepsilon_{ij}$$

$$\varepsilon_{ij} \sim N(0, \sigma_\varepsilon^2)$$

$$u_{0i} \sim N(0, \sigma_u^2)$$

where $i = 1, ...1018$ indexes the children in ARIES, $j = 1,2,3$ indexes the measurement occasion and $a_+ = a$ if $a > 0$ or 0 otherwise. β_1 gives the average difference in methylation of offspring of normal weight and obese mothers; β_2 gives the average change in methylation from birth to adolescence; β_3 tells us whether there is any change to this trend (i.e. β_2) from childhood to adolescence; β_4 tells us whether there is a difference in methylation change between obese mother- and normal weight mother- offspring; and β_5 tells us whether the offspring of obese and normal weight mothers have a *different* change to the trend (i.e. β_2) of methylation change from birth to childhood. From these we can calculate the change in methylation from 0-7 for children of normal weight mothers (β_2), obese mothers ($\beta_2 + \beta_4$) and the change from 7-17 for children of normal weight mothers ($\beta_2 + \beta_3$) and obese mothers ($\beta_2 + \beta_3 + \beta_4 + \beta_5$). To test whether there is a difference in methylation change between 7 and 17 we test whether $\beta_4 + \beta_5$ is different from zero, and present a p-value for this in our results.

For each CpG site, we used a multilevel model, adjusting for confounders (offspring sex, maternal age, parity, smoking status and occupation) and the first 20 independent surrogate variable components (which account for cellular heterogeneity between the cord blood and whole blood cells). To correct for multiple testing, across the CpG sites and two parameters of (difference in change during childhood/adolescence) interest we used a cut-off of 0.05/(2*number of CpG sites), which for the obese comparison was 8.9×10^{-4} and for the underweight comparison was 1.5×10^{-5}.

1. Laird N, Ware J. Random-effects for longitudinal data. Biometrics. 1982;38(4):963–974.

2. Goldstein H. Multilevel mixed linear model analysis using iterative generalized least squares. Biometrika. 1986 Apr 1;73(1):43–56.

Tables and figures

Table S1. Comparison of maternal baseline characteristics in ALSPAC mothers included and not included in ARIES.

Characteristic	ALSPAC mothers included in ARIES	ALSPAC mothers not included in ARIES
	Mean (SD) , Median (IQR) or %*	Mean (SD) , Median (IQR) or %*
**Reported pre-pregnancy BMI**	(n=944)	(n=10633)
	22.8 (3.7)	22.9 (3.8)
**Pregnancy stage-specific GWG**	(n=971)	(n=11512)
0 to 18 weeks (kg/wk)	0.3 (0.2)	0.3 (0.2)
18 to 28 weeks (kg/wk)	0.5 (0.2)	0.5 (0.2)
28 weeks to delivery (kg/wk)	0.5 (0.2)	0.5 (0.2)
**Total GWG (kg)**	(n=939)	(n=11486)
	12.6 (4.4)	12.5 (4.8)
**IoM categories of GWG**	(n=881)	(n=9401)
Below recommended GWG	322	3146
Recommended GWG	334	3671
Over recommended GWG	225	2584
**Offspring sex**	(n=1018)	(n=13041)
Male	48.8%	51.9%
Female	51.2%	48.1%
**Gestational age at delivery (weeks)**	(n=1018)	(n=13614)
	40 (39 – 41)	40 (39 – 41)
**Parity**	(n=989)	(n=12002)
Nulliparous	46.4%	44.6%
Multiparous	53.6%	55.5%
**Age at delivery (years)**	(n=986)	(n=10838)
	29.6 (4.4)	28.2 (4.8)
**Occupation**	(n=901)	(n=9188)
Manual occupation	14.0%	20.5%
Non-manual occupation	86.0%	79.5%
**Smoking status**	(n=1006)	(n=12166)
Never before or during pregnancy	86.7%	73.3%
Before pregnancy or during 1st trimester only	3.6%	7.3%
Throughout pregnancy	9.7%	19.4%

*SD = standard deviation; IQR = interquartile range

Table S2. Associations between maternal and offspring adiposity in ARIES (mean difference (95% confidence interval)).

Offspring outcome	Maternal exposure						
	Pre-pregnancy BMI (kg/m^2)[a]	Underweight (compared to normal weight) [a]	Overweight (compared to normal weight) [a]	Obese (compared to normal weight) [a]	GWG in early pregnancy (400g/wk) [b]	GWG in mid-pregnancy (400g/wk) [c]	GWG in late pregnancy (400g/wk) [d]
Birth weight (g)[e]	15.0 (6.7 to 23.3)	-142.7 (-304.4 to 18.9)	50.5 (-39.2 to 140.2)	191.5 (46.8 to 336.1)	168.0 (96.8 to 239.1)	208.9 (129.6 to 288.3)	112.1 (-28.7 to 195.5)
BMI at age 7 (kg/m^2) [f]	0.2 (0.1 to 0.2)	-0.9 (-1.6 to -0.3)	0.7 (0.3 to 1.1)	2.5 (1.8 to 3.1)	0.03 (-0.3 to 0.4)	0.3(-0.1 to 0.6)	0.7 (0.3 to 1.1)
Waist circumference at age 7 (cm) [f]	0.3 (0.2 to 0.4)	-1.7 (-3.1 to -0.3)	1.3 (0.4 to 2.1)	4.7 (3.3 to 6.1)	0.1 (-0.8 to 0.6)	-0.3 (-1.1 to 0.6)	1.4 (0.6 to 2.2)
Lean mass at age 9 (g) [f]	111.9 (78.4 to 145.3)	-510.1 (-1115.4 to 95.1)	620.4 (262.0 to 978.8)	1281.7 (696.8 to 1866.6)	-143.4 (-434.0 to 147.3)	-213.9 (-544.87 to 117.11)	279.8 (-61.6 to 621.2)
Fat mass at age 9 (g) [f]	390.8 (309.2 to 472.5)	-1338.3 (-2761.9 to 85.2)	2200.3 (1335.5 to 3065.1)	5068.9 (3663.8 to 6474.1)	305.3 (-415.5 to 1026.1)	143.0 (-678.7 to 964.6)	1166.2 (321.6 to 2010.8)
BMI at age 15 (kg/m^2) [f]	0.4 (0.3 to 0.4)	-1.8 (-2.9 to 0.6)	2.0 (1.3 to 2.6)	4.3 (3.2 to 5.4)	0.03 (-0.5 to 0.6)	0.4 (-0.2 to 1.1)	0.9 (0.2 to 1.5)
Waist circumference at age 15 (cm) [f]	0.8 (0.7 to 1.0)	-3.1 (-6.5 to 0.4)	4.0 (2.2 to 5.8)	11.0 (8.0 to 14.0)	-0.7 (-2.2 to 0.8)	0.5 (-1.2 to 2.2)	1.1 (-0.7 to 3.0)
Lean mass at age 15 (g) [f]	223.1 (143.4 to 302.9)	-1013.9 (-2588.6 to 560.7)	1039.8 (199.6 to 1880.1)	2224.3 (821.8 to 3626.9)	132.3 (-565.3 to 829.9)	123.2 (-675.9 to 922.2)	560.6 (-260.1 to 1381.4)
Fat mass at age 15 (g) [f]	775.1 (621.9 to 928.4)	-3704.7 (-6494.8 to -914.7)	4334.0 (2757.9 to 5910.2)	9503.8 (6919.9 to 12087.6)	-28.6 (-1372.5 to 1315.3)	1156.5 (-380.5 to 2693.6)	1811.6 (236.7 to 3386.5)

[a] Adjusted for offspring sex, maternal age, maternal parity, maternal smoking status and maternal occupation

[b] Adjusted for offspring sex, maternal BMI, maternal age, maternal parity, maternal smoking status and maternal occupation

[c] Adjusted for offspring sex, maternal BMI, GWG in early pregnancy, maternal age, maternal parity, maternal smoking status and maternal occupation

[d] Adjusted for offspring sex, maternal BMI, GWG in early pregnancy, GWG in mid-pregnancy, maternal age, maternal parity, maternal smoking status and maternal occupation

[e] Additionally adjusted for gestational age at delivery

[f] Additionally adjusted for age (months) and height (cm) at measurement

Table S3. Associations between maternal adiposity and estimated cell-type proportion (mean difference (95% confidence interval)).

Outcome	Pre-pregnancy BMI (kg/m2)	Pre-pregnancy obesity (vs. normal weight)	Pre-pregnancy overweight (vs. normal weight)	Pre-pregnancy underweight (vs. normal weight)	GWG in early pregnancy (400g/week)	GWG in mid-pregnancy (400g/week)	GWG in late pregnancy (400g/week)
B-cells	0.05 (-0.01 to 0.12)	0.22 (-0.93 to 1.37)	1.37 (0.65 to 2.08)	-0.07 (-1.41 to 1.27)	0.18 (-0.40 to 0.76)	0.43 (-0.14 to 1.00)	0.39 (-0.12 to 0.90)
CD4 T-cells	-0.11 (-0.20 to -0.02)	-0.67 (-2.27 to 0.93)	-0.73 (-1.68 to 0.23)	0.77 (-1.04 to 2.58)	-0.01 (-0.79 to 0.78)	0.03 (-0.74 to 0.80)	-0.10 (-0.78 to 0.59)
CD8 T-cells	-0.02 (-0.10 to 0.06)	-0.45 (-1.81 to 0.92)	0.04 (-0.81 to 0.89)	-0.16 (-1.75 to 1.43)	-0.23 (-0.91 to 0.46)	-0.71 (-1.38 to -0.03)	-0.49 (-1.09 to 0.11)
Granulocytes	0.09 (-0.06 to 0.23)	1.65 (-0.90 to 4.20)	-1.12 (-2.69 to 0.45)	-0.69 (-3.66 to 2.29)	0.10 (-1.18 to 1.37)	-0.58 (-1.83 to 0.67)	-0.32 (-1.43 to 0.80)
Monocytes	0.06 (0.01 to 0.11)	0.37 (-0.51 to 1.25)	0.68 (0.12 to 1.24)	-0.28 (-1.32 to 0.75)	0.04 (-0.40 to 0.49)	0.33 (-0.11 to 0.77)	0.40 (0.01 to 0.79)
NK cells	-0.01 (-0.08 to 0.06)	-0.68 (-1.91 to 0.55)	0.52 (-0.25 to 1.30)	0.04 (-1.40 to 1.47)	0.16 (-0.47 to 0.78)	0.61 (0.00 to 1.22)	0.33 (-0.22 to 0.87)

Table S4. Comparison of the number of CpG sites identified by each regression model in epigenome-wide association studies of maternal adiposity and offspring cord blood DNA methylation (FDR-adjusted P-value < 0.05).

Exposure (n for Model 2)	Number of cord blood CpG sites identified with FDR-adjusted P<0.1 (and over effect size cut-off)		
	Model 1[c]	Model 2[d]	Model 3[e]
Maternal pre-pregnancy BMI (n=727)	0	2	0
Maternal pre-pregnancy underweight (n=24[a])	1066	1621	1793
Maternal pre-pregnancy overweight (n=94[a])	0	0	0
Maternal pre-pregnancy obesity (n=32[a])	42	28	38
GWG in early pregnancy (n=690)	0	0	0
GWG in mid-pregnancy (n=690)	0	0	0
GWG in late pregnancy (n=690)	0	0	0
Total GWG (n=673)	0	0	0
Under IOM-recommended GWG (n=242[b])	0	0	0
Over IOM-recommended GWG (n=170[b])	0	0	0

[a]compared to normal range BMI (n=577)
[b] compared to IOM-recommended range (n=258)
[c] adjusted for bisulfite conversion batch
[d] adjusted for bisulfite conversion batch and covariates
[f] adjusted for bisulfite conversion batch, covariates and estimated cell-type proportions.

Table S5. Comparison of our results to associations of maternal adiposity with cord blood methylation that have been reported in the literature.

Gene [a]	Author	Exposure in the previously published study	Exposure in our study	Number of probes on 450k array	Number of probes in filtered array [b]	Direction of change reported in the previously published study	Number of probes negatively associated with the exposure in our study	Range of P-values for negatively associated probes	Number of probes positively associated with the exposure in our study	Range of P-values for positively associated probes
ZCCHC10 (cg01422136)	Liu et al. (2014)	Maternal pre-pregnancy BMI	Maternal pre-pregnancy BMI	1	1	Negative	1	0.58	0	n/a
MMP7	Morales et al. (2014)	Gestational weight gain in early pregnancy	Gestational weight gain in early pregnancy	7	5	Positive	4	0.12 to 0.71	1	0.82
RXRA	Godfrey et al. (2011)	Lower maternal carbohydrate intake in early pregnancy	Maternal pre-pregnancy underweight	44	36	Positive	24	0.03 to 0.99	12	0.01 to 0.92
PPARGC1A	Gemma et al. (2012)	Maternal pre-pregnancy BMI	Maternal pre-pregnancy BMI	18	11	Positive	6	0.004 to 0.92	5	0.28 to 0.99

[a] Where the published study did not use the 450k assay, we used the gene name rather than the probe ID.

[b] Filtered array refers to the set of probes we used to conduct our analyses (i.e. 450k array minus probes listed in: Naeem et al. BMC Genomics. 2014 Jan 22;15(1):51, probes with a high detection P-value and non-autosomal probes)

Table S6. Percentage of sites where the direction of association is the same for the "maternal adiposity-offspring methylation" relationship and the "offspring methylation-offspring adiposity" relationship.

	Maternal obesity associated with greater methylation (i.e. greater BMI associated with greater methylation) [n sites = 22]	Maternal obesity associated with lower methylation (i.e. greater BMI associated with lower methylation) [n sites = 6]	Maternal underweight associated with greater methylation (i.e. greater BMI associated with lower methylation) [n sites = 1425]	Maternal underweight associated with lower methylation (i.e. greater BMI associated with greater methylation) [n sites = 196]
Birth weight	81.82%	83.33%	78.74%	75.00%
BMI at age 7	68.18%	50.00%	70.88%	66.33%
BMI at age 15	77.30%	50.00%	55.65%	57.14%
Waist circumference at age 7	63.64%	83.33%	30.32%	40.31%
Waist circumference at age 15	68.18%	100%	51.65%	60.20%
Fat mass at age 9	63.64%	100%	44.98%	46.43%
Fat mass at age 15	81.82%	50.00%	52.14%	51.02%
Lean mass at age 9	72.73%	50.00%	20.28%	24.49%
Lean mass at age 15	68.18%	83.33%	39.58%	49.49%

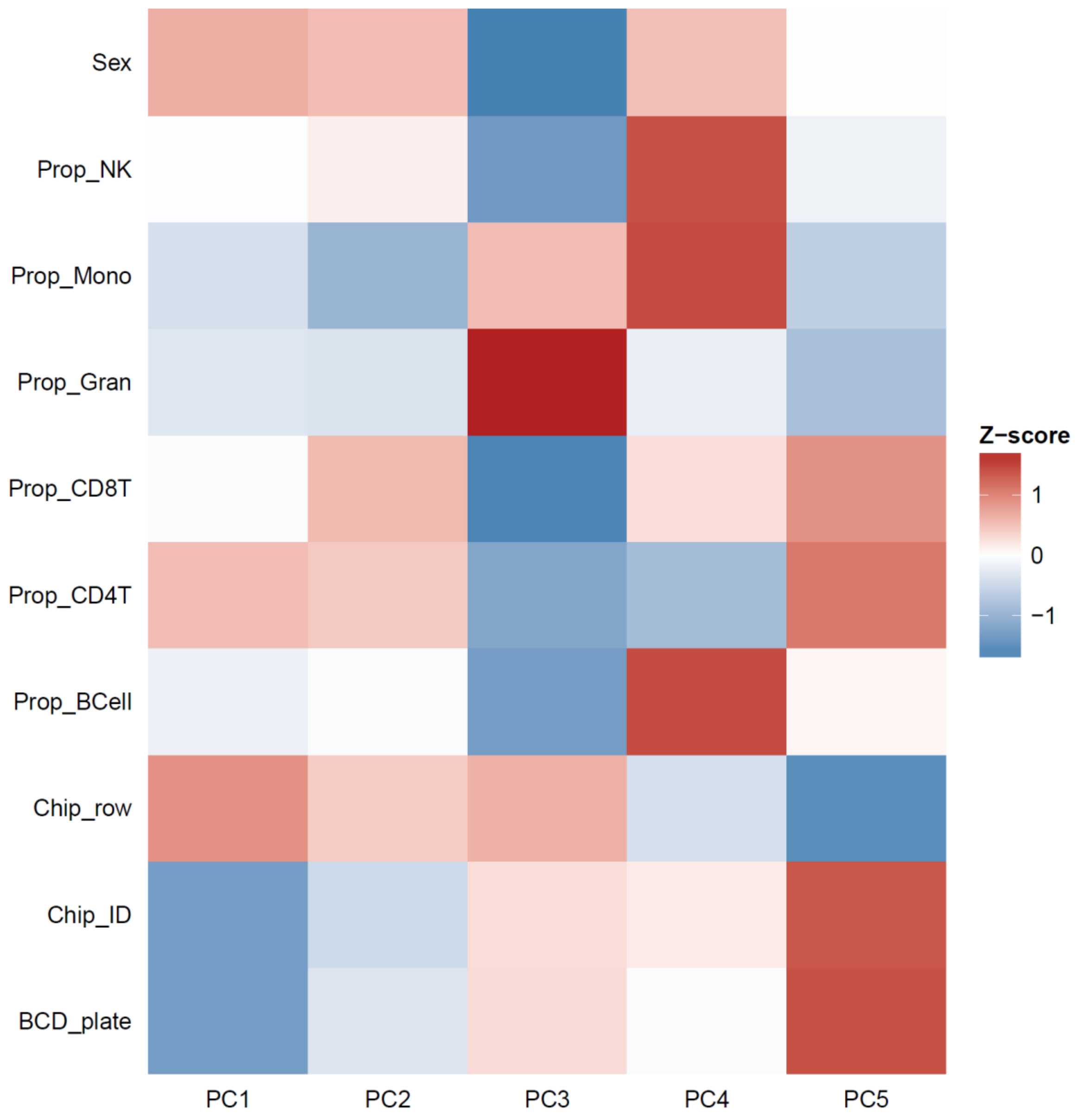

Figure S1. A heatmap to show the effect estimates of associations between different batch variables (BCD_plate (bisulfite-conversion batch); Chip_ID Chip_row), cell type proportions (B cell, CD4-T cells, CD8-T cells, granulocytes, monocytes and natural killer (NK) cells, sex and principal components for cord blood DNA methylation. BCD_plate was identified as the major batch variable in ARIES. Slide level batch (Chip_ID) adjustment is less useful because samples from all time points in ARIES were distributed across slides using a semi-random approach (sampling criteria were in place to ensure that all time points were represented on each array), therefore, (for any time point) each slide (chip) will contain only a small number of samples. Allocation to BCD_plate is more likely to contain systematic bias, as samples were added to the batch according to laboratory priorities and convenience.

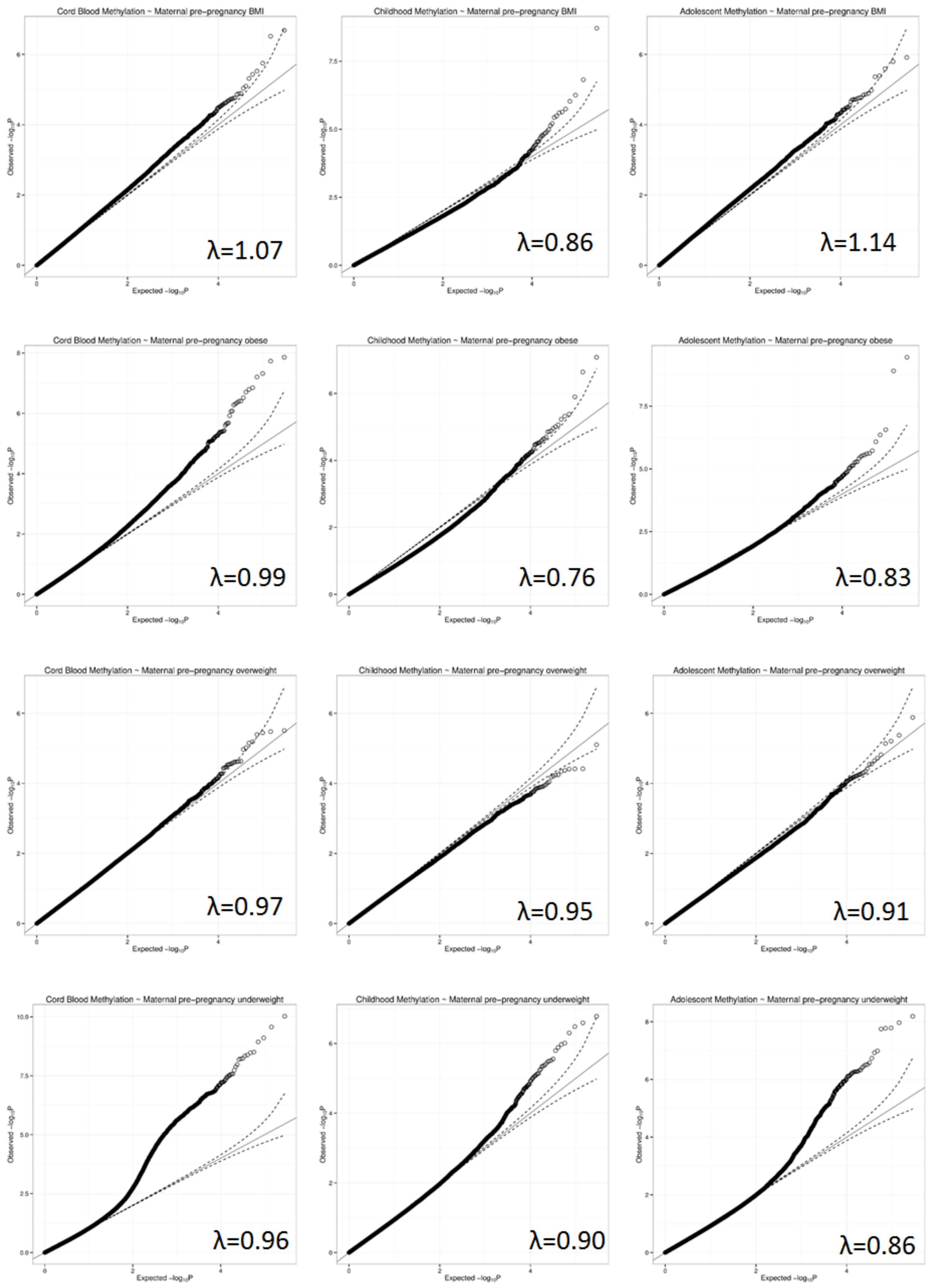
Cord Blood Methylation ~ Maternal pre-pregnancy BMI
λ=1.07
Childhood Methylation ~ Maternal pre-pregnancy BMI
λ=0.86
Adolescent Methylation ~ Maternal pre-pregnancy BMI
λ=1.14
Cord Blood Methylation ~ Maternal pre-pregnancy obese
λ=0.99
Childhood Methylation ~ Maternal pre-pregnancy obese
λ=0.76
Adolescent Methylation ~ Maternal pre-pregnancy obese
λ=0.83
Cord Blood Methylation ~ Maternal pre-pregnancy overweight
λ=0.97
Childhood Methylation ~ Maternal pre-pregnancy overweight
λ=0.95
Adolescent Methylation ~ Maternal pre-pregnancy overweight
λ=0.91
Cord Blood Methylation ~ Maternal pre-pregnancy underweight
λ=0.96
Childhood Methylation ~ Maternal pre-pregnancy underweight
λ=0.90
Adolescent Methylation ~ Maternal pre-pregnancy underweight
λ=0.86
Expected $-\log_{10}P$
Observed $-\log_{10}P$

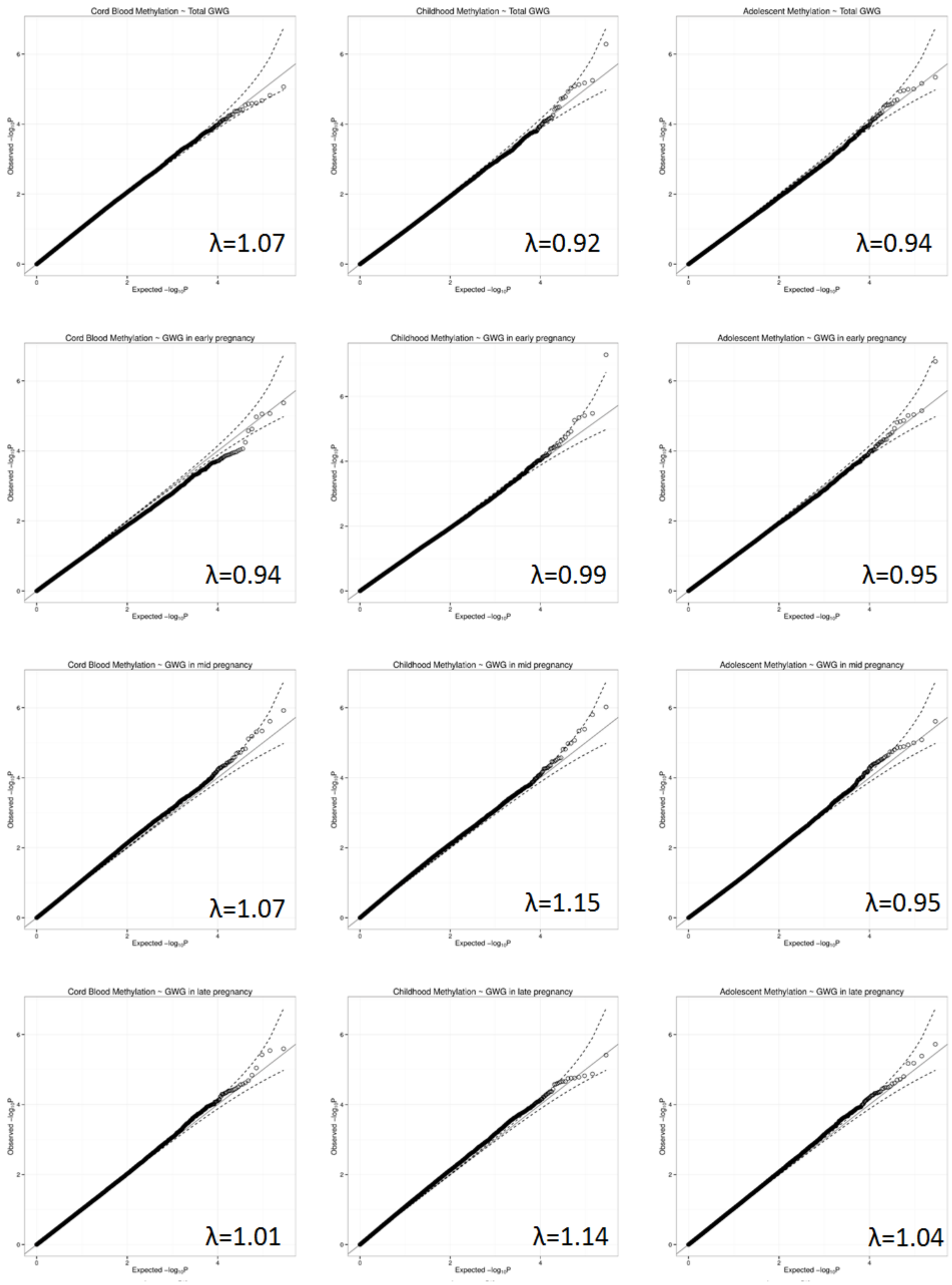
Cord Blood Methylation ~ Total GWG
λ=1.07
Childhood Methylation ~ Total GWG
λ=0.92
Adolescent Methylation ~ Total GWG
λ=0.94
Cord Blood Methylation ~ GWG in early pregnancy
λ=0.94
Childhood Methylation ~ GWG in early pregnancy
λ=0.99
Adolescent Methylation ~ GWG in early pregnancy
λ=0.95
Cord Blood Methylation ~ GWG in mid pregnancy
λ=1.07
Childhood Methylation ~ GWG in mid pregnancy
λ=1.15
Adolescent Methylation ~ GWG in mid pregnancy
λ=0.95
Cord Blood Methylation ~ GWG in late pregnancy
λ=1.01
Childhood Methylation ~ GWG in late pregnancy
λ=1.14
Adolescent Methylation ~ GWG in late pregnancy
λ=1.04
Expected −log10P
Observed −log10P

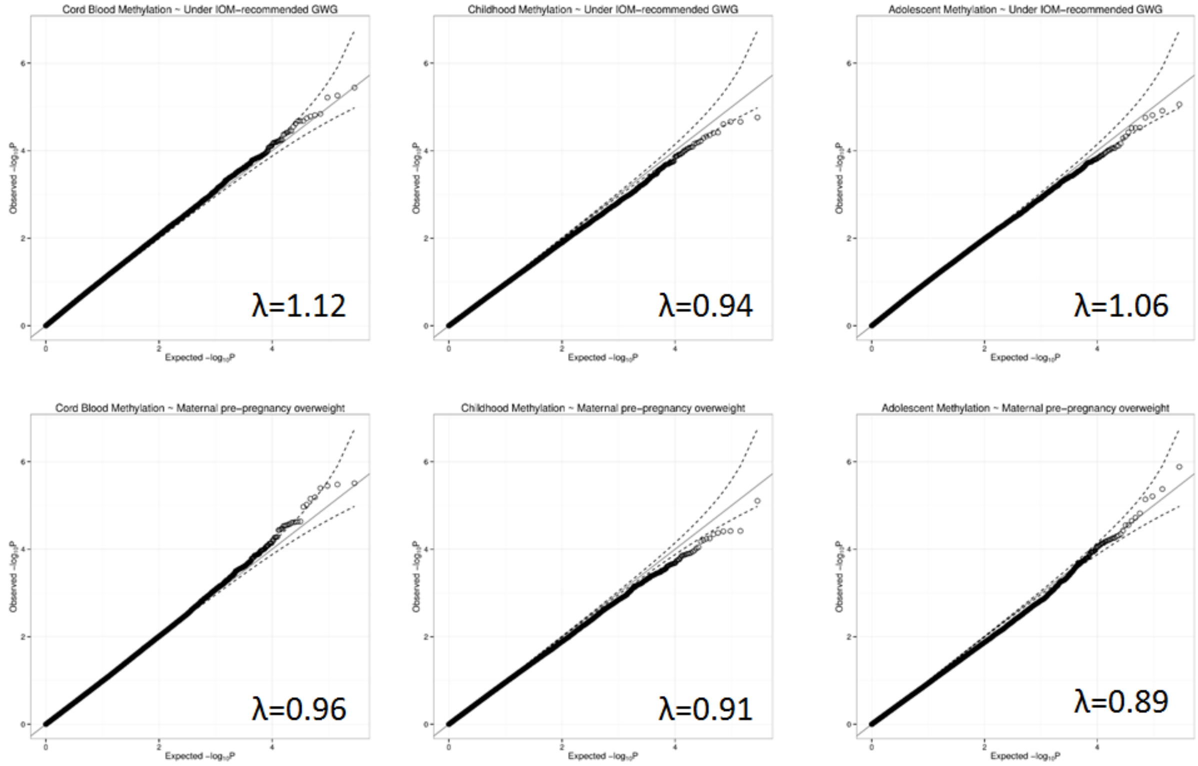

Figure S2. Quantile-quantile (Q-Q) plots with genomic inflation values (Lambdas) for each EWAS.
